# Supplementary figures and images for: Combining plasma extracellular vesicle Let-7b-5p, miR-184 and circulating miR-22-3p levels for NSCLC diagnosis and drug resistance prediction
Source: Sci Rep. 2022 Apr 23;12:6693. doi: 10.1038/s41598-022-10598-x (PMC9035169; doi:10.1038/s41598-022-10598-x)

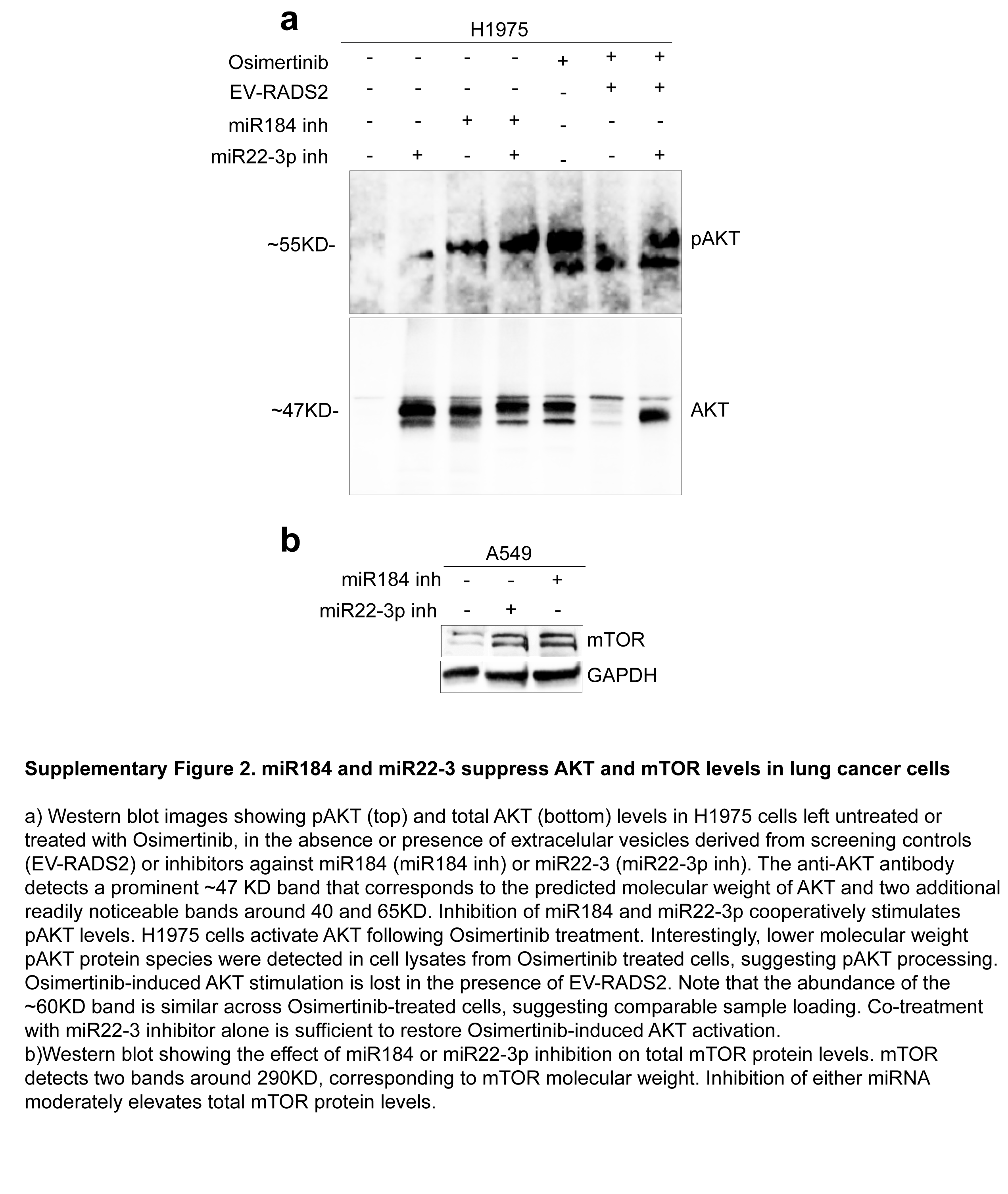

Supplement: Supplementary file 3 — Supplementary Figure 2. [file 41598_2022_10598_MOESM3_ESM.tif]
