# Supplementary material for: Combining plasma extracellular vesicle Let-7b-5p, miR-184 and circulating miR-22-3p levels for NSCLC diagnosis and drug resistance prediction
Source: Sci Rep. 2022 Apr 23;12:6693. doi: 10.1038/s41598-022-10598-x (PMC9035169; doi:10.1038/s41598-022-10598-x)
Supplement: Supplementary file 5 — Supplementary Table 2. [file 41598_2022_10598_MOESM5_ESM.pdf]

Cut-point	Sensitivity%	95% CI	Specificity%	95% CI
> 0.08341	100,0	86,20% to 100,0%	9,091	0,4663% to 37,74%
> 0.1044	100,0	86,20% to 100,0%	18,18	3,231% to 47,70%
> 0.1454	100,0	86,20% to 100,0%	27,27	9,746% to 56,56%
> 0.1759	100,0	86,20% to 100,0%	36,36	15,17% to 64,62%
> 0.2095	100,0	86,20% to 100,0%	45,45	21,27% to 71,99%
> 0.2545	95,83	79,76% to 99,79%	45,45	21,27% to 71,99%
> 0.2770	95,83	79,76% to 99,79%	54,55	28,01% to 78,73%
> 0.2879	95,83	79,76% to 99,79%	63,64	35,38% to 84,83%
> 0.3212	95,83	79,76% to 99,79%	72,73	43,44% to 90,25%
> 0.3927	91,67	74,15% to 98,52%	72,73	43,44% to 90,25%
> 0.4872	87,50	69,00% to 95,66%	72,73	43,44% to 90,25%
> 0.5451	87,50	69,00% to 95,66%	81,82	52,30% to 96,77%
> 0.5765	83,33	64,15% to 93,32%	81,82	52,30% to 96,77%
> 0.6931	79,17	59,53% to 90,76%	81,82	52,30% to 96,77%
> 0.7977	75,00	55,10% to 88,00%	81,82	52,30% to 96,77%
> 0.8115	70,83	50,83% to 85,09%	81,82	52,30% to 96,77%
> 0.8178	70,83	50,83% to 85,09%	90,91	62,26% to 99,53%
> 0.8601	70,83	50,83% to 85,09%	100,0	74,12% to 100,0%
> 0.9021	66,67	46,71% to 82,03%	100,0	74,12% to 100,0%
> 0.9170	62,50	42,71% to 78,84%	100,0	74,12% to 100,0%
> 0.9338	58,33	38,83% to 75,53%	100,0	74,12% to 100,0%
> 0.9460	54,17	35,07% to 72,11%	100,0	74,12% to 100,0%
> 0.9718	50,00	31,43% to 68,57%	100,0	74,12% to 100,0%
> 0.9889	45,83	27,89% to 64,93%	100,0	74,12% to 100,0%
> 0.9898	41,67	24,47% to 61,17%	100,0	74,12% to 100,0%
> 0.9912	37,50	21,16% to 57,29%	100,0	74,12% to 100,0%
> 0.9935	33,33	17,97% to 53,29%	100,0	74,12% to 100,0%
> 0.9946	29,17	14,91% to 49,17%	100,0	74,12% to 100,0%
> 0.9954	25,00	12,00% to 44,90%	100,0	74,12% to 100,0%
> 0.9967	20,83	9,245% to 40,47%	100,0	74,12% to 100,0%
> 0.9982	16,67	6,679% to 35,85%	100,0	74,12% to 100,0%
> 0.9994	12,50	4,344% to 31,00%	100,0	74,12% to 100,0%
> 0.9998	8,333	1,481% to 25,85%	100,0	74,12% to 100,0%
> 0.9999	4,167	0,2137% to 20,24%	100,0	74,12% to 100,0%

Area under the ROC curve	
Area	0,9242
Std. Error	0,04442
95% confidence interval	0,8372 to 1,000
P value	<0,0001
Data	
Controls (P (negative))	11
Patients (P (positive))	24
Missing Controls	0
Missing Patients	2

Supplementary table 2. Parameters and summary of combined let-7b-5p, miR-184, and miR-22-3p receiver operating characteristic analyses. Cut-point, sensitivity, specificities, and 95% confidence interval values are presented.
