# Supplementary material for: Combining plasma extracellular vesicle Let-7b-5p, miR-184 and circulating miR-22-3p levels for NSCLC diagnosis and drug resistance prediction
Source: Sci Rep. 2022 Apr 23;12:6693. doi: 10.1038/s41598-022-10598-x (PMC9035169; doi:10.1038/s41598-022-10598-x)
Supplement: Supplementary file 6 — Supplementary Table 3. [file 41598_2022_10598_MOESM6_ESM.pdf]

miRNA	Accession	Target	Target ID	Experiment	Literature
hsa-let-7b-5p	MIMAT0000063	ABCF1	23	CLASH	23622248
hsa-let-7b-5p	MIMAT0000063	ABL1	25	CLASH	23622248
hsa-let-7b-5p	MIMAT0000063	ACACA	31	CLASH//Proteomics	18668040|23622248
hsa-let-7b-5p	MIMAT0000063	ASIC1	41	CLASH	23622248
hsa-let-7b-5p	MIMAT0000063	ACPP	55	Proteomics	18668040
hsa-let-7b-5p	MIMAT0000063	ACTA1	58	PAR-CLIP	21572407
hsa-let-7b-5p	MIMAT0000063	ACTB	60	CLASH	23622248
hsa-let-7b-5p	MIMAT0000063	ACTG1	71	CLASH//Luciferase reporter assay//Reporter assay	15131085|23622248
hsa-let-7b-5p	MIMAT0000063	ACTN4	81	CLASH	23622248
hsa-let-7b-5p	MIMAT0000063	ACVR1	90	Luciferase reporter assay//Microarray//qRT-PCR	22995917
hsa-let-7b-5p	MIMAT0000063	ADCY1	107	CLASH	23622248
hsa-let-7b-5p	MIMAT0000063	ADH5	128	HITS-CLIP//PAR-CLIP	23313552|26701625|27292025
hsa-let-7b-5p	MIMAT0000063	AGL	178	Proteomics	18668040
hsa-let-7b-5p	MIMAT0000063	JAG1	182	CLASH	23622248
hsa-let-7b-5p	MIMAT0000063	AHR	196	PAR-CLIP	23592263
hsa-let-7b-5p	MIMAT0000063	AK4	205	PAR-CLIP	23446348|20371350
hsa-let-7b-5p	MIMAT0000063	AKT2	208	Luciferase reporter assay// qRT-PCR//Western blot	25288334
hsa-let-7b-5p	MIMAT0000063	AMD1	262	HITS-CLIP	23313552
hsa-let-7b-5p	MIMAT0000063	AMPH	273	CLASH	23622248
hsa-let-7b-5p	MIMAT0000063	SLC25A4	291	CLASH	23622248
hsa-let-7b-5p	MIMAT0000063	BIRC5	332	CLASH	23622248
hsa-let-7b-5p	MIMAT0000063	APRT	353	Proteomics	18668040
hsa-let-7b-5p	MIMAT0000063	AQP6	363	PAR-CLIP	27292025
hsa-let-7b-5p	MIMAT0000063	AR	367	CLASH	23622248
hsa-let-7b-5p	MIMAT0000063	ARCN1	372	Proteomics	18668040
hsa-let-7b-5p	MIMAT0000063	RHOB	388	Proteomics//pSILAC	18668040
hsa-let-7b-5p	MIMAT0000063	RHOG	391	Proteomics//pSILAC	18668040
hsa-let-7b-5p	MIMAT0000063	ASNA1	439	CLASH	23622248

hsa-let-7b-5p	MIMAT0000063	ASPA	443	CLASH	23622248
hsa-let-7b-5p	MIMAT0000063	ATOX1	475	CLASH	23622248
hsa-let-7b-5p	MIMAT0000063	ATP1A1	476	CLASH	23622248
hsa-let-7b-5p	MIMAT0000063	ATP2A2	488	CLASH//Proteomics	18668040|23622248
hsa-let-7b-5p	MIMAT0000063	ALDH7A1	501	CLASH	23622248
hsa-let-7b-5p	MIMAT0000063	ATP6V0A1	535	Proteomics//pSILAC	18668040
hsa-let-7b-5p	MIMAT0000063	AUP1	550	CLASH	23622248
hsa-let-7b-5p	MIMAT0000063	BACH1	571	PAR-CLIP	20371350
hsa-let-7b-5p	MIMAT0000063	BCAT1	586	CLASH	23622248
hsa-let-7b-5p	MIMAT0000063	CCND1	595	Annexin V-FITC//immunoblot//Immunoblot//Immunofluorescence//Luciferase reporter assay//Microarray//Northern blot//PAR-CLIP//qRT-PCR//Reporter assay//Western blot	18379589|20133835|23806108|23592263|26701625
hsa-let-7b-5p	MIMAT0000063	BCL7A	605	Microarray//qRT-PCR	18026111
hsa-let-7b-5p	MIMAT0000063	BFSP1	631	CLASH	23622248
hsa-let-7b-5p	MIMAT0000063	BGLAP	632	Proteomics	18668040
hsa-let-7b-5p	MIMAT0000063	PRDM1	639	Immunohistochemistry//Luciferase reporter assay//qRT-PCR//Western blot	20651244
hsa-let-7b-5p	MIMAT0000063	BMP7	655	CLASH	23622248
hsa-let-7b-5p	MIMAT0000063	POLR3D	661	HITS-CLIP	23313552
hsa-let-7b-5p	MIMAT0000063	BNIP3L	665	CLASH	23622248
hsa-let-7b-5p	MIMAT0000063	VPS51	738	Proteomics	18668040
hsa-let-7b-5p	MIMAT0000063	CA12	771	Proteomics	18668040
hsa-let-7b-5p	MIMAT0000063	CALU	813	PAR-CLIP	20371350
hsa-let-7b-5p	MIMAT0000063	CAPG	822	Proteomics//pSILAC	18668040
hsa-let-7b-5p	MIMAT0000063	CBFB	865	Microarray	17699775
hsa-let-7b-5p	MIMAT0000063	CCNA2	890	Immunoblot//Immunofluorescence//Luciferase reporter assay//qRT-PCR	18379589
hsa-let-7b-5p	MIMAT0000063	CCNB1	891	CLASH	23622248

hsa-let-7b-5p	MIMAT0000063	CCND2	894	Immunohistochemistry//Luciferase reporter assay//qRT-PCR//QRTPCR//Western blot	17699775|17942906|23482325
hsa-let-7b-5p	MIMAT0000063	CCND3	896	CLASH	23622248
hsa-let-7b-5p	MIMAT0000063	CCNF	899	CLASH//Microarray	17699775|23622248
hsa-let-7b-5p	MIMAT0000063	CCNG1	900	CLASH	23622248
hsa-let-7b-5p	MIMAT0000063	CCNT2	905	PAR-CLIP	21572407
hsa-let-7b-5p	MIMAT0000063	ENTPD6	955	CLASH	23622248
hsa-let-7b-5p	MIMAT0000063	CD59	966	PAR-CLIP	23446348|20371350
hsa-let-7b-5p	MIMAT0000063	CD81	975	CLASH	23622248
hsa-let-7b-5p	MIMAT0000063	CD151	977	CLASH	23622248
hsa-let-7b-5p	MIMAT0000063	CDC25A	993	Immunohistochemistry//Luciferase reporter assay//Microarray//qRT-PCR//Western blot	19966857|17699775
hsa-let-7b-5p	MIMAT0000063	CDC34	997	Immunoblot//Luciferase reporter assay//Microarray//qRT-PCR//Western blot	19126550|17699775|21252116
hsa-let-7b-5p	MIMAT0000063	CDK6	1021	Luciferase reporter assay//Microarray//Western blot	17699775
hsa-let-7b-5p	MIMAT0000063	CDKN1A	1026	Immunoblot//Microarray//PAR-CLIP//qRT-PCR	21572407|25578966
hsa-let-7b-5p	MIMAT0000063	CDKN1B	1027	Immunoblot//Microarray//qRT-PCR	25578966
hsa-let-7b-5p	MIMAT0000063	CENPB	1059	Proteomics	18668040
hsa-let-7b-5p	MIMAT0000063	CHD1	1105	Proteomics	18668040
hsa-let-7b-5p	MIMAT0000063	CHD3	1107	CLASH//Proteomics	18668040|23622248
hsa-let-7b-5p	MIMAT0000063	CHD4	1108	CLASH//Proteomics	18668040|23622248
hsa-let-7b-5p	MIMAT0000063	CKB	1152	CLASH	23622248
hsa-let-7b-5p	MIMAT0000063	CKS2	1164	CLASH	23622248
hsa-let-7b-5p	MIMAT0000063	AP1S1	1174	PAR-CLIP	23592263|26701625
hsa-let-7b-5p	MIMAT0000063	COL3A1	1281	Luciferase reporter assay//Microarray//qRT-PCR	22995917
hsa-let-7b-5p	MIMAT0000063	COL8A1	1295	PAR-CLIP	23592263

hsa-let-7b-5p	MIMAT0000063	COX6B1	1340	HITS-CLIP	23706177
hsa-let-7b-5p	MIMAT0000063	COX7B	1349	Proteomics	18668040
hsa-let-7b-5p	MIMAT0000063	CRKL	1399	Proteomics	18668040
hsa-let-7b-5p	MIMAT0000063	CRX	1406	PAR-CLIP	26701625
hsa-let-7b-5p	MIMAT0000063	CRY2	1408	PAR-CLIP	23592263|26701625
hsa-let-7b-5p	MIMAT0000063	CS	1431	Proteomics	18668040
hsa-let-7b-5p	MIMAT0000063	CSNK1D	1453	Proteomics//pSILAC	18668040
hsa-let-7b-5p	MIMAT0000063	CSNK2A1	1457	CLASH	23622248
hsa-let-7b-5p	MIMAT0000063	CTBP2	1488	CLASH	23622248
hsa-let-7b-5p	MIMAT0000063	CTPS1	1503	HITS-CLIP//Proteomics	18668040|23313552
hsa-let-7b-5p	MIMAT0000063	CUX1	1523	CLASH	23622248
hsa-let-7b-5p	MIMAT0000063	CYP1A2	1544	CLASH	23622248
hsa-let-7b-5p	MIMAT0000063	CYP2J2	1573	Luciferase reporter assay//qRT-PCR//Western blot	22761738
hsa-let-7b-5p	MIMAT0000063	DCTD	1635	CLASH	23622248
hsa-let-7b-5p	MIMAT0000063	DHX9	1660	CLASH	23622248
hsa-let-7b-5p	MIMAT0000063	DDX10	1662	Proteomics	18668040
hsa-let-7b-5p	MIMAT0000063	DFFA	1676	CLASH	23622248
hsa-let-7b-5p	MIMAT0000063	TIMM8A	1678	Proteomics	18668040
hsa-let-7b-5p	MIMAT0000063	DIAPH1	1729	CLASH//Proteomics	18668040|23622248
hsa-let-7b-5p	MIMAT0000063	DLAT	1737	Proteomics	18668040
hsa-let-7b-5p	MIMAT0000063	DMD	1756	Microarray	17699775
hsa-let-7b-5p	MIMAT0000063	DNA2	1763	PAR-CLIP	23446348
hsa-let-7b-5p	MIMAT0000063	DNAH9	1770	HITS-CLIP	23706177
hsa-let-7b-5p	MIMAT0000063	DYNC1H1	1778	CLASH	23622248
hsa-let-7b-5p	MIMAT0000063	DRG2	1819	Proteomics	18668040
hsa-let-7b-5p	MIMAT0000063	ARID3A	1820	Microarray//PAR-CLIP	17699775|23592263
hsa-let-7b-5p	MIMAT0000063	RCAN1	1827	Microarray	17699775
hsa-let-7b-5p	MIMAT0000063	DSG2	1829	Proteomics	18668040
hsa-let-7b-5p	MIMAT0000063	DSP	1832	CLASH//Proteomics//pSILAC	18668040|23622248
hsa-let-7b-5p	MIMAT0000063	DUSP1	1843	PAR-CLIP	21572407
hsa-let-7b-5p	MIMAT0000063	DVL3	1857	PAR-CLIP	23446348
hsa-let-7b-5p	MIMAT0000063	E2F2	1870	Immunofluorescence//Luciferase reporter assay//qRT-PCR//Western blot	27520092

hsa-let-7b-5p	MIMAT0000063	E2F3	1871	CLASH	23622248
hsa-let-7b-5p	MIMAT0000063	E2F5	1875	Microarray	17699775
hsa-let-7b-5p	MIMAT0000063	E2F6	1876	Microarray//PAR-CLIP	17699775|20371350
hsa-let-7b-5p	MIMAT0000063	EDN1	1906	PAR-CLIP	23592263
hsa-let-7b-5p	MIMAT0000063	EEF1A1	1915	CLASH	23622248
hsa-let-7b-5p	MIMAT0000063	EEF2	1938	CLASH	23622248
hsa-let-7b-5p	MIMAT0000063	EIF4A1	1973	Proteomics	18668040
hsa-let-7b-5p	MIMAT0000063	EIF4A2	1974	Proteomics	18668040
hsa-let-7b-5p	MIMAT0000063	EIF4G2	1982	PAR-CLIP//Proteomics	18668040|23592263|21572407|27292025
hsa-let-7b-5p	MIMAT0000063	ELK4	2005	CLASH	23622248
hsa-let-7b-5p	MIMAT0000063	ENG	2022	Proteomics	18668040
hsa-let-7b-5p	MIMAT0000063	EP300	2033	CLASH	23622248
hsa-let-7b-5p	MIMAT0000063	EPHA4	2043	PAR-CLIP	21572407
hsa-let-7b-5p	MIMAT0000063	ERCC1	2067	CLASH	23622248
hsa-let-7b-5p	MIMAT0000063	ETFA	2108	CLASH	23622248
hsa-let-7b-5p	MIMAT0000063	EZH2	2146	qRT-PCR//Western blot	25611389
hsa-let-7b-5p	MIMAT0000063	F2	2147	Proteomics	18668040
hsa-let-7b-5p	MIMAT0000063	FANCD2	2177	CLASH	23622248
hsa-let-7b-5p	MIMAT0000063	ACSL1	2180	Proteomics	18668040
hsa-let-7b-5p	MIMAT0000063	FEN1	2237	CLASH	23622248
hsa-let-7b-5p	MIMAT0000063	FLII	2314	CLASH	23622248
hsa-let-7b-5p	MIMAT0000063	FLNA	2316	CLASH	23622248
hsa-let-7b-5p	MIMAT0000063	FMO4	2329	HITS-CLIP	23824327
hsa-let-7b-5p	MIMAT0000063	FPR1	2357	HITS-CLIP	23313552
hsa-let-7b-5p	MIMAT0000063	FXN	2395	PAR-CLIP	23592263
hsa-let-7b-5p	MIMAT0000063	GABPB1	2553	HITS-CLIP//PAR-CLIP	23592263|23313552
hsa-let-7b-5p	MIMAT0000063	GALNT2	2590	Proteomics	18668040
hsa-let-7b-5p	MIMAT0000063	GAPDH	2597	CLASH	23622248
hsa-let-7b-5p	MIMAT0000063	GATA6	2627	CLASH	23622248
hsa-let-7b-5p	MIMAT0000063	GATM	2628	PAR-CLIP	26701625
hsa-let-7b-5p	MIMAT0000063	NR6A1	2649	PAR-CLIP	23592263
hsa-let-7b-5p	MIMAT0000063	GLB1	2720	Proteomics	18668040
hsa-let-7b-5p	MIMAT0000063	GLO1	2739	PAR-CLIP//Proteomics	18668040|23592263
hsa-let-7b-5p	MIMAT0000063	GNAS	2778	CLASH	23622248
hsa-let-7b-5p	MIMAT0000063	GNB1	2782	CLASH	23622248
hsa-let-7b-5p	MIMAT0000063	GNG5	2787	PAR-CLIP	20371350|27292025
hsa-let-7b-5p	MIMAT0000063	GOLGA4	2803	PAR-CLIP	20371350
hsa-let-7b-5p	MIMAT0000063	GPI	2821	CLASH	23622248

hsa-let-7b-5p	MIMAT0000063	GPM6B	2824	CLASH	23622248
hsa-let-7b-5p	MIMAT0000063	GPX7	2882	Microarray	17699775
hsa-let-7b-5p	MIMAT0000063	GSK3A	2931	CLASH	23622248
hsa-let-7b-5p	MIMAT0000063	GSPT1	2935	Proteomics	18668040
hsa-let-7b-5p	MIMAT0000063	GSR	2936	CLASH//Proteomics	18668040|23622248
hsa-let-7b-5p	MIMAT0000063	GTF2I	2969	Microarray	17699775
hsa-let-7b-5p	MIMAT0000063	GTF3C1	2975	CLASH	23622248
hsa-let-7b-5p	MIMAT0000063	GYG1	2992	CLASH	23622248
hsa-let-7b-5p	MIMAT0000063	GYS1	2997	Proteomics//pSILAC	18668040
hsa-let-7b-5p	MIMAT0000063	GYS2	2998	Proteomics	18668040
hsa-let-7b-5p	MIMAT0000063	HIST1H1C	3006	CLASH	23622248
hsa-let-7b-5p	MIMAT0000063	HIST1H2BD	3017	PAR-CLIP	24398324|23446348
hsa-let-7b-5p	MIMAT0000063	HADHA	3030	CLASH	23622248
hsa-let-7b-5p	MIMAT0000063	HARS	3035	CLASH	23622248
hsa-let-7b-5p	MIMAT0000063	HCFC1	3054	CLASH	23622248
hsa-let-7b-5p	MIMAT0000063	HTT	3064	CLASH	23622248
hsa-let-7b-5p	MIMAT0000063	HELLS	3070	Proteomics	18668040
hsa-let-7b-5p	MIMAT0000063	HIF1A	3091	CLASH	23622248
hsa-let-7b-5p	MIMAT0000063	HK1	3098	CLASH	23622248
hsa-let-7b-5p	MIMAT0000063	HMGB1	3146	CLASH	23622248
hsa-let-7b-5p	MIMAT0000063	HMGCS1	3157	CLASH	23622248
hsa-let-7b-5p	MIMAT0000063	HMGA1	3159	CLASH//HITS-CLIP//Luciferase reporter assay//Microarray//PAR-CLIP//Proteomics//pSILAC//qRT-PCR//Western blot	18668040|23622248|23798998|23592263|23313552
hsa-let-7b-5p	MIMAT0000063	HNRNPF	3185	CLASH	23622248
hsa-let-7b-5p	MIMAT0000063	HNRNPL	3191	CLASH	23622248
hsa-let-7b-5p	MIMAT0000063	HOXD11	3237	CLASH	23622248
hsa-let-7b-5p	MIMAT0000063	HRAS	3265	Immunoblot//qRT-PCR//Western blot	21252116
hsa-let-7b-5p	MIMAT0000063	AGFG2	3268	CLASH	23622248
hsa-let-7b-5p	MIMAT0000063	HES1	3280	CLASH	23622248
hsa-let-7b-5p	MIMAT0000063	HSF2	3298	CLASH	23622248
hsa-let-7b-5p	MIMAT0000063	HSPA1B	3304	CLASH	23622248
hsa-let-7b-5p	MIMAT0000063	HSPA8	3312	CLASH	23622248
hsa-let-7b-5p	MIMAT0000063	HSP90AA1	3320	CLASH	23622248

hsa-let-7b-5p	MIMAT0000063	IGSF3	3321	Proteomics	18668040
hsa-let-7b-5p	MIMAT0000063	IDI1	3422	Proteomics	18668040
hsa-let-7b-5p	MIMAT0000063	IFNB1	3456	ELISA//Luciferase reporter assay//qRT-PCR	20130213
hsa-let-7b-5p	MIMAT0000063	IFRD1	3475	Proteomics//pSILAC	18668040
hsa-let-7b-5p	MIMAT0000063	IGF1R	3480	Immunohistochemistry//Luciferase reporter assay//PAR-CLIP//QRTPCR//Western blot	23482325|23592263|24398324|23446348|21572407
hsa-let-7b-5p	MIMAT0000063	IGHMBP2	3508	CLASH	23622248
hsa-let-7b-5p	MIMAT0000063	RBPJ	3516	CLASH	23622248
hsa-let-7b-5p	MIMAT0000063	IL6R	3570	PAR-CLIP	27292025
hsa-let-7b-5p	MIMAT0000063	CXCL8	3576	PAR-CLIP	26701625
hsa-let-7b-5p	MIMAT0000063	IMPDH1	3614	Proteomics	18668040
hsa-let-7b-5p	MIMAT0000063	IMPDH2	3615	Proteomics	18668040
hsa-let-7b-5p	MIMAT0000063	INPPL1	3636	Proteomics	18668040
hsa-let-7b-5p	MIMAT0000063	ITGA3	3675	HITS-CLIP	23706177|23313552
hsa-let-7b-5p	MIMAT0000063	ITGB5	3693	CLASH	23622248
hsa-let-7b-5p	MIMAT0000063	KCNC4	3749	CLASH	23622248
hsa-let-7b-5p	MIMAT0000063	KIF2A	3796	Proteomics	18668040
hsa-let-7b-5p	MIMAT0000063	KIFC1	3833	CLASH	23622248
hsa-let-7b-5p	MIMAT0000063	KPNA5	3841	HITS-CLIP//PAR-CLIP	24398324|23446348|21572407|20371350
hsa-let-7b-5p	MIMAT0000063	TNPO1	3842	CLASH	23622248
hsa-let-7b-5p	MIMAT0000063	RPSA	3921	CLASH	23622248
hsa-let-7b-5p	MIMAT0000063	LBR	3930	Proteomics	18668040
hsa-let-7b-5p	MIMAT0000063	LDLR	3949	Proteomics	18668040
hsa-let-7b-5p	MIMAT0000063	LPL	4023	Proteomics	18668040
hsa-let-7b-5p	MIMAT0000063	LTA4H	4048	CLASH	23622248
hsa-let-7b-5p	MIMAT0000063	LYN	4067	PAR-CLIP	23592263
hsa-let-7b-5p	MIMAT0000063	MAB21L1	4081	CLASH	23622248
hsa-let-7b-5p	MIMAT0000063	MXD1	4084	PAR-CLIP	21572407
hsa-let-7b-5p	MIMAT0000063	MAGEA3	4102	PAR-CLIP	27292025
hsa-let-7b-5p	MIMAT0000063	MAGEA6	4105	PAR-CLIP	27292025
hsa-let-7b-5p	MIMAT0000063	MAGEA12	4111	PAR-CLIP	27292025
hsa-let-7b-5p	MIMAT0000063	MAP4	4134	CLASH	23622248
hsa-let-7b-5p	MIMAT0000063	MARS	4141	Proteomics	18668040

hsa-let-7b-5p	MIMAT0000063	MCM4	4173	CLASH	23622248
hsa-let-7b-5p	MIMAT0000063	MCM7	4176	CLASH	23622248
hsa-let-7b-5p	MIMAT0000063	MDM4	4194	CLASH//PAR-CLIP	23622248|23592263|24398324
hsa-let-7b-5p	MIMAT0000063	MEF2C	4208	CLASH	23622248
hsa-let-7b-5p	MIMAT0000063	MEF2D	4209	PAR-CLIP	26701625
hsa-let-7b-5p	MIMAT0000063	MEIS3P1	4213	PAR-CLIP	23592263
hsa-let-7b-5p	MIMAT0000063	CD99	4267	CLASH	23622248
hsa-let-7b-5p	MIMAT0000063	MIPEP	4285	Proteomics	18668040
hsa-let-7b-5p	MIMAT0000063	MLLT1	4298	Proteomics//pSILAC	18668040
hsa-let-7b-5p	MIMAT0000063	MOV10	4343	CLASH	23622248
hsa-let-7b-5p	MIMAT0000063	MPG	4350	Proteomics	18668040
hsa-let-7b-5p	MIMAT0000063	ABCC1	4363	Proteomics	18668040
hsa-let-7b-5p	MIMAT0000063	MSI1	4440	CLASH	23622248
hsa-let-7b-5p	MIMAT0000063	MSN	4478	CLASH	23622248
hsa-let-7b-5p	MIMAT0000063	ATP6	4508	CLASH	23622248
hsa-let-7b-5p	MIMAT0000063	COX1	4512	CLASH	23622248
hsa-let-7b-5p	MIMAT0000063	COX2	4513	CLASH	23622248
hsa-let-7b-5p	MIMAT0000063	COX3	4514	CLASH	23622248
hsa-let-7b-5p	MIMAT0000063	ND1	4535	CLASH	23622248
hsa-let-7b-5p	MIMAT0000063	ND2	4536	CLASH	23622248
hsa-let-7b-5p	MIMAT0000063	ND4	4538	CLASH	23622248
hsa-let-7b-5p	MIMAT0000063	ND4L	4539	CLASH	23622248
hsa-let-7b-5p	MIMAT0000063	ND5	4540	CLASH	23622248
hsa-let-7b-5p	MIMAT0000063	MTRR	4552	Proteomics//pSILAC	18668040
hsa-let-7b-5p	MIMAT0000063	MYC	4609	CLASH//TRAP	23622248|24510096
hsa-let-7b-5p	MIMAT0000063	MYO1C	4641	CLASH	23622248
hsa-let-7b-5p	MIMAT0000063	MYO1E	4643	Proteomics	18668040
hsa-let-7b-5p	MIMAT0000063	NACA	4666	CLASH	23622248
hsa-let-7b-5p	MIMAT0000063	NAP1L1	4673	HITS-CLIP	19536157
hsa-let-7b-5p	MIMAT0000063	NDUFA10	4705	CLASH	23622248
hsa-let-7b-5p	MIMAT0000063	NEDD4	4734	Proteomics//pSILAC	18668040
hsa-let-7b-5p	MIMAT0000063	NFATC1	4772	CLASH	23622248
hsa-let-7b-5p	MIMAT0000063	NFATC3	4775	CLASH	23622248
hsa-let-7b-5p	MIMAT0000063	NFKBIA	4792	CLASH	23622248
hsa-let-7b-5p	MIMAT0000063	NME4	4833	CLASH	23622248
hsa-let-7b-5p	MIMAT0000063	CNOT2	4848	CLASH	23622248
hsa-let-7b-5p	MIMAT0000063	SLC11A2	4891	HITS-CLIP//PAR-CLIP	23592263|23313552

hsa-let-7b-5p	MIMAT0000063	NRAS	4893	Immunohistochemistry//Luciferase reporter assay//Proteomics//qRT-PCR	17699775|19966857|18668040
hsa-let-7b-5p	MIMAT0000063	NRDC	4898	CLASH	23622248
hsa-let-7b-5p	MIMAT0000063	NUCB2	4925	PAR-CLIP	23592263
hsa-let-7b-5p	MIMAT0000063	NVL	4931	Proteomics	18668040
hsa-let-7b-5p	MIMAT0000063	OPRL1	4987	PAR-CLIP	22012620
hsa-let-7b-5p	MIMAT0000063	ORC4	5000	Proteomics	18668040
hsa-let-7b-5p	MIMAT0000063	OXA1L	5018	Proteomics	18668040
hsa-let-7b-5p	MIMAT0000063	PA2G4	5036	CLASH	23622248
hsa-let-7b-5p	MIMAT0000063	PAFAH1B3	5050	Proteomics	18668040
hsa-let-7b-5p	MIMAT0000063	PAFAH2	5051	HITS-CLIP	19536157
hsa-let-7b-5p	MIMAT0000063	PAK1	5058	CLASH	23622248
hsa-let-7b-5p	MIMAT0000063	PAX3	5077	CLASH	23622248
hsa-let-7b-5p	MIMAT0000063	PBX2	5089	PAR-CLIP	26701625
hsa-let-7b-5p	MIMAT0000063	PCBP2	5094	CLASH	23622248
hsa-let-7b-5p	MIMAT0000063	PCCB	5096	Proteomics	18668040
hsa-let-7b-5p	MIMAT0000063	PCYT1A	5130	Proteomics	18668040
hsa-let-7b-5p	MIMAT0000063	PDGFB	5155	PAR-CLIP	27292025
hsa-let-7b-5p	MIMAT0000063	PDGFRA	5156	Luciferase reporter assay//Microarray//qRT-PCR	17942906|22995917
hsa-let-7b-5p	MIMAT0000063	PDK1	5163	Proteomics	18668040
hsa-let-7b-5p	MIMAT0000063	PER1	5187	CLASH	23622248
hsa-let-7b-5p	MIMAT0000063	PEX6	5190	CLASH	23622248
hsa-let-7b-5p	MIMAT0000063	PFKM	5213	CLASH	23622248
hsa-let-7b-5p	MIMAT0000063	PFN1	5216	CLASH	23622248
hsa-let-7b-5p	MIMAT0000063	PGM1	5236	CLASH	23622248
hsa-let-7b-5p	MIMAT0000063	PGM3	5238	Proteomics	18668040
hsa-let-7b-5p	MIMAT0000063	PHKA1	5255	CLASH	23622248
hsa-let-7b-5p	MIMAT0000063	PLAGL2	5326	Microarray//PAR-CLIP	17699775|23592263|26701625
hsa-let-7b-5p	MIMAT0000063	PLCB3	5331	Proteomics	18668040
hsa-let-7b-5p	MIMAT0000063	PLCG2	5336	PAR-CLIP	24398324|23446348
hsa-let-7b-5p	MIMAT0000063	PLK1	5347	CLASH	23622248
hsa-let-7b-5p	MIMAT0000063	PMAIP1	5366	PAR-CLIP	23592263
hsa-let-7b-5p	MIMAT0000063	Sep-04	5414	Proteomics	18668040
hsa-let-7b-5p	MIMAT0000063	POLD2	5425	Proteomics//pSILAC	18668040
hsa-let-7b-5p	MIMAT0000063	POLR2A	5430	CLASH	23622248

hsa-let-7b-5p	MIMAT0000063	POLR2C	5432	Proteomics//pSILAC	18668040
hsa-let-7b-5p	MIMAT0000063	POLR2D	5433	PAR-CLIP	23592263|21572407|26701625
hsa-let-7b-5p	MIMAT0000063	POLR2H	5437	Proteomics	18668040
hsa-let-7b-5p	MIMAT0000063	POLR2L	5441	Proteomics	18668040
hsa-let-7b-5p	MIMAT0000063	PPID	5481	Proteomics	18668040
hsa-let-7b-5p	MIMAT0000063	PPM1G	5496	Proteomics	18668040
hsa-let-7b-5p	MIMAT0000063	PPP1R7	5510	Proteomics//pSILAC	18668040
hsa-let-7b-5p	MIMAT0000063	PPP2R2A	5520	CLASH//PAR-CLIP	23622248|23446348
hsa-let-7b-5p	MIMAT0000063	PPP2R5E	5529	CLASH	23622248
hsa-let-7b-5p	MIMAT0000063	PRIM1	5557	Proteomics//pSILAC	18668040
hsa-let-7b-5p	MIMAT0000063	PRIM2	5558	HITS-CLIP	23706177
hsa-let-7b-5p	MIMAT0000063	PRKAA2	5563	CLASH	23622248
hsa-let-7b-5p	MIMAT0000063	PRKAR2<br>A	5576	Proteomics	18668040
hsa-let-7b-5p	MIMAT0000063	PRKD1	5587	CLASH	23622248
hsa-let-7b-5p	MIMAT0000063	MAPK1	5594	CLASH	23622248
hsa-let-7b-5p	MIMAT0000063	MAPK6	5597	PAR-CLIP	21572407
hsa-let-7b-5p	MIMAT0000063	MAP2K2	5605	Proteomics	18668040
hsa-let-7b-5p	MIMAT0000063	MAP2K7	5609	PAR-CLIP	23592263|26701625
hsa-let-7b-5p	MIMAT0000063	PRPS1	5631	Proteomics	18668040
hsa-let-7b-5p	MIMAT0000063	PSMD9	5715	Proteomics	18668040
hsa-let-7b-5p	MIMAT0000063	PTGFRN	5738	CLASH	23622248
hsa-let-7b-5p	MIMAT0000063	PTGS2	5743	Proteomics//pSILAC	18668040
hsa-let-7b-5p	MIMAT0000063	PVR	5817	Proteomics	18668040
hsa-let-7b-5p	MIMAT0000063	PCYT2	5833	CLASH	23622248
hsa-let-7b-5p	MIMAT0000063	QARS	5859	Proteomics	18668040
hsa-let-7b-5p	MIMAT0000063	QDPR	5860	PAR-CLIP	23592263
hsa-let-7b-5p	MIMAT0000063	RALB	5899	CLASH	23622248
hsa-let-7b-5p	MIMAT0000063	RBBP6	5930	CLASH	23622248
hsa-let-7b-5p	MIMAT0000063	RDX	5962	PAR-CLIP//Proteomics	18668040|23446348|21572407|2670162<br>5
hsa-let-7b-5p	MIMAT0000063	DPF2	5977	Proteomics	18668040
hsa-let-7b-5p	MIMAT0000063	RFC2	5982	HITS-CLIP	23706177
hsa-let-7b-5p	MIMAT0000063	RHD	6007	HITS-CLIP//PAR-CLIP	23592263|23706177|23313552
hsa-let-7b-5p	MIMAT0000063	BRD2	6046	CLASH	23622248
hsa-let-7b-5p	MIMAT0000063	RPL12	6136	CLASH	23622248
hsa-let-7b-5p	MIMAT0000063	RPL18	6141	CLASH	23622248
hsa-let-7b-5p	MIMAT0000063	RPL18A	6142	CLASH	23622248
hsa-let-7b-5p	MIMAT0000063	MRPL12	6182	PAR-CLIP	23592263
hsa-let-7b-5p	MIMAT0000063	RPS4X	6191	CLASH	23622248

hsa-let-7b-5p	MIMAT0000063	RPS24	6229	CLASH	23622248
hsa-let-7b-5p	MIMAT0000063	RRAD	6236	PAR-CLIP	22012620
hsa-let-7b-5p	MIMAT0000063	RRBP1	6238	CLASH	23622248
hsa-let-7b-5p	MIMAT0000063	RRM1	6240	PAR-CLIP	23592263
hsa-let-7b-5p	MIMAT0000063	RRM2	6241	Microarray//PAR-CLIP//Proteomics	17699775|18668040|21572407
hsa-let-7b-5p	MIMAT0000063	RXRB	6257	CLASH	23622248
hsa-let-7b-5p	MIMAT0000063	SAFB	6294	CLASH	23622248
hsa-let-7b-5p	MIMAT0000063	SALL2	6297	CLASH	23622248
hsa-let-7b-5p	MIMAT0000063	SC5D	6309	CLASH	23622248
hsa-let-7b-5p	MIMAT0000063	ATXN2	6311	CLASH//HITS-CLIP	23622248|23313552
hsa-let-7b-5p	MIMAT0000063	SCD	6319	CLASH//Proteomics	18668040|23622248
hsa-let-7b-5p	MIMAT0000063	SEMG2	6407	Proteomics	18668040
hsa-let-7b-5p	MIMAT0000063	SKI	6497	CLASH	23622248
hsa-let-7b-5p	MIMAT0000063	SLC1A4	6509	Proteomics//pSILAC	18668040
hsa-let-7b-5p	MIMAT0000063	SLC20A1	6574	PAR-CLIP	23592263|20371350
hsa-let-7b-5p	MIMAT0000063	SLC25A1	6576	Proteomics	18668040
hsa-let-7b-5p	MIMAT0000063	SMARCA1	6594	Proteomics	18668040
hsa-let-7b-5p	MIMAT0000063	SMARCA4	6597	CLASH//Proteomics	18668040|23622248
hsa-let-7b-5p	MIMAT0000063	SMARCB1	6598	CLASH	23622248
hsa-let-7b-5p	MIMAT0000063	SMARCC1	6599	Proteomics	18668040
hsa-let-7b-5p	MIMAT0000063	SMARCC2	6601	CLASH	23622248
hsa-let-7b-5p	MIMAT0000063	SMARCD1	6602	CLASH//Proteomics	18668040|23622248
hsa-let-7b-5p	MIMAT0000063	SUMO2	6613	CLASH	23622248
hsa-let-7b-5p	MIMAT0000063	SNRPA	6626	CLASH	23622248
hsa-let-7b-5p	MIMAT0000063	SNRPE	6635	CLASH	23622248
hsa-let-7b-5p	MIMAT0000063	SOD2	6648	PAR-CLIP	22012620
hsa-let-7b-5p	MIMAT0000063	SON	6651	CLASH	23622248
hsa-let-7b-5p	MIMAT0000063	SOX9	6662	Microarray	17699775
hsa-let-7b-5p	MIMAT0000063	SP1	6667	CLASH	23622248
hsa-let-7b-5p	MIMAT0000063	SP100	6672	Proteomics	18668040
hsa-let-7b-5p	MIMAT0000063	SPN	6693	CLASH	23622248
hsa-let-7b-5p	MIMAT0000063	SPR	6697	Proteomics	18668040
hsa-let-7b-5p	MIMAT0000063	SPTBN2	6712	CLASH	23622248
hsa-let-7b-5p	MIMAT0000063	TROVE2	6738	Proteomics	18668040
hsa-let-7b-5p	MIMAT0000063	SSR1	6745	Microarray	17699775
hsa-let-7b-5p	MIMAT0000063	ST13	6767	CLASH	23622248

hsa-let-7b-5p	MIMAT0000063	STAT2	6773	HITS-CLIP	23706177
hsa-let-7b-5p	MIMAT0000063	STIM1	6786	Proteomics	18668040
hsa-let-7b-5p	MIMAT0000063	STK4	6789	PAR-CLIP	23446348
hsa-let-7b-5p	MIMAT0000063	AURKA	6790	Microarray//Proteomics	17699775|18668040
hsa-let-7b-5p	MIMAT0000063	STRN	6801	PAR-CLIP	24398324|20371350
hsa-let-7b-5p	MIMAT0000063	STX3	6809	PAR-CLIP	27292025
hsa-let-7b-5p	MIMAT0000063	SUOX	6821	PAR-CLIP	21572407
hsa-let-7b-5p	MIMAT0000063	SURF4	6836	PAR-CLIP	20371350
hsa-let-7b-5p	MIMAT0000063	SYT1	6857	PAR-CLIP	26701625
hsa-let-7b-5p	MIMAT0000063	TAF9	6880	Proteomics	18668040
hsa-let-7b-5p	MIMAT0000063	TCOF1	6949	Proteomics	18668040
hsa-let-7b-5p	MIMAT0000063	TGFBR1	7046	ELISA//GFP reporter assay//Western blot	24978044
hsa-let-7b-5p	MIMAT0000063	TGFBR3	7049	PAR-CLIP	21572407
hsa-let-7b-5p	MIMAT0000063	THBS1	7057	PAR-CLIP//Proteomics//pSILAC	18668040|24398324
hsa-let-7b-5p	MIMAT0000063	TIAM1	7074	CLASH	23622248
hsa-let-7b-5p	MIMAT0000063	TJP1	7082	CLASH	23622248
hsa-let-7b-5p	MIMAT0000063	TLN1	7094	CLASH	23622248
hsa-let-7b-5p	MIMAT0000063	TLR4	7099	Luciferase reporter assay	23437218
hsa-let-7b-5p	MIMAT0000063	NR2E1	7101	immunoblot//Luciferase reporter assay//Northern blot//Western blot	20133835
hsa-let-7b-5p	MIMAT0000063	TRAPPC10	7109	PAR-CLIP	26701625
hsa-let-7b-5p	MIMAT0000063	TPBG	7162	Proteomics	18668040
hsa-let-7b-5p	MIMAT0000063	TPD52L2	7165	CLASH	23622248
hsa-let-7b-5p	MIMAT0000063	TPM4	7171	CLASH	23622248
hsa-let-7b-5p	MIMAT0000063	TPP2	7174	Proteomics	18668040
hsa-let-7b-5p	MIMAT0000063	TPT1	7178	CLASH	23622248
hsa-let-7b-5p	MIMAT0000063	TST	7263	Proteomics	18668040
hsa-let-7b-5p	MIMAT0000063	TUBB2A	7280	PAR-CLIP	23592263
hsa-let-7b-5p	MIMAT0000063	TYMS	7298	Proteomics//pSILAC	18668040
hsa-let-7b-5p	MIMAT0000063	UBA1	7317	CLASH	23622248
hsa-let-7b-5p	MIMAT0000063	UBE2A	7319	CLASH	23622248
hsa-let-7b-5p	MIMAT0000063	UBE2D2	7322	Proteomics	18668040
hsa-let-7b-5p	MIMAT0000063	UBE2D3	7323	Proteomics	18668040
hsa-let-7b-5p	MIMAT0000063	UBE2I	7329	Proteomics	18668040
hsa-let-7b-5p	MIMAT0000063	SUMO1	7341	PAR-CLIP	24398324

hsa-let-7b-5p	MIMAT0000063	UGT8	7368	Proteomics//pSILAC	18668040
hsa-let-7b-5p	MIMAT0000063	UCK2	7371	Proteomics	18668040
hsa-let-7b-5p	MIMAT0000063	UTRN	7402	CLASH	23622248
hsa-let-7b-5p	MIMAT0000063	VCL	7414	PAR-CLIP	23592263
hsa-let-7b-5p	MIMAT0000063	YWHAE	7531	CLASH	23622248
hsa-let-7b-5p	MIMAT0000063	YWHAZ	7534	CLASH//PAR-CLIP	23622248|23592263
hsa-let-7b-5p	MIMAT0000063	ZNF3	7551	CLASH	23622248
hsa-let-7b-5p	MIMAT0000063	ZNF8	7554	PAR-CLIP	23592263
hsa-let-7b-5p	MIMAT0000063	ZNF28	7576	PAR-CLIP	23592263
hsa-let-7b-5p	MIMAT0000063	ZNF148	7707	CLASH	23622248
hsa-let-7b-5p	MIMAT0000063	ZMYM2	7750	CLASH	23622248
hsa-let-7b-5p	MIMAT0000063	ZNF200	7752	PAR-CLIP	21572407|20371350
hsa-let-7b-5p	MIMAT0000063	ZNF207	7756	CLASH	23622248
hsa-let-7b-5p	MIMAT0000063	SLC30A1	7779	Proteomics	18668040
hsa-let-7b-5p	MIMAT0000063	BTG2	7832	CLASH	23622248
hsa-let-7b-5p	MIMAT0000063	PXDN	7837	Proteomics//pSILAC	18668040
hsa-let-7b-5p	MIMAT0000063	BRPF1	7862	CLASH	23622248
hsa-let-7b-5p	MIMAT0000063	PRRC2A	7916	CLASH//Proteomics//pSILAC	18668040|23622248
hsa-let-7b-5p	MIMAT0000063	BAG6	7917	CLASH	23622248
hsa-let-7b-5p	MIMAT0000063	ARHGEF5	7984	CLASH	23622248
hsa-let-7b-5p	MIMAT0000063	UBXN8	7993	Proteomics	18668040
hsa-let-7b-5p	MIMAT0000063	NUP214	8021	CLASH//Proteomics	18668040|23622248
hsa-let-7b-5p	MIMAT0000063	MLLT10	8028	PAR-CLIP	23592263|27292025
hsa-let-7b-5p	MIMAT0000063	PDHX	8050	Proteomics	18668040
hsa-let-7b-5p	MIMAT0000063	PTP4A2	8073	CLASH	23622248
hsa-let-7b-5p	MIMAT0000063	KMT2D	8085	PAR-CLIP	24398324|21572407
hsa-let-7b-5p	MIMAT0000063	HMGA2	8091	HITS-CLIP//Immunohistochemistry//Luciferase reporter assay//Microarray//PAR-CLIP//qRT-PCR//QRTPCR//Western blot	17437991|23482325|23318420|20371350|21572407|25600877|26701625
hsa-let-7b-5p	MIMAT0000063	COIL	8161	PAR-CLIP//Proteomics	18668040|23446348|21572407|20371350
hsa-let-7b-5p	MIMAT0000063	NCOA3	8202	PAR-CLIP	24398324|26701625
hsa-let-7b-5p	MIMAT0000063	SMC1A	8243	CLASH//PAR-CLIP	23622248|23446348|21572407|20371350|26701625

hsa-let-7b-5p	MIMAT0000063	NAA10	8260	CLASH	23622248
hsa-let-7b-5p	MIMAT0000063	SLC10A3	8273	CLASH	23622248
hsa-let-7b-5p	MIMAT0000063	FZD9	8326	PAR-CLIP	23446348|21572407
hsa-let-7b-5p	MIMAT0000063	HIST1H3B	8358	CLASH	23622248
hsa-let-7b-5p	MIMAT0000063	DYRK3	8444	PAR-CLIP	26701625
hsa-let-7b-5p	MIMAT0000063	DHX16	8449	Proteomics	18668040
hsa-let-7b-5p	MIMAT0000063	CUL3	8452	Proteomics	18668040
hsa-let-7b-5p	MIMAT0000063	CUL2	8453	CLASH	23622248
hsa-let-7b-5p	MIMAT0000063	CUL1	8454	Proteomics	18668040
hsa-let-7b-5p	MIMAT0000063	IRS4	8471	CLASH	23622248
hsa-let-7b-5p	MIMAT0000063	DENR	8562	CLASH	23622248
hsa-let-7b-5p	MIMAT0000063	THOC5	8563	CLASH	23622248
hsa-let-7b-5p	MIMAT0000063	KHSRP	8570	CLASH	23622248
hsa-let-7b-5p	MIMAT0000063	NOP14	8602	Proteomics	18668040
hsa-let-7b-5p	MIMAT0000063	SLC25A12	8604	Proteomics	18668040
hsa-let-7b-5p	MIMAT0000063	USO1	8615	Proteomics	18668040
hsa-let-7b-5p	MIMAT0000063	RTCA	8634	CLASH//Proteomics	18668040|23622248
hsa-let-7b-5p	MIMAT0000063	SOCS1	8651	PAR-CLIP	23592263
hsa-let-7b-5p	MIMAT0000063	IRS2	8660	CLASH//Flow//Luciferase reporter assay//qRT-PCR//Western blot	23622248|24810113
hsa-let-7b-5p	MIMAT0000063	EIF3C	8663	CLASH	23622248
hsa-let-7b-5p	MIMAT0000063	EIF3D	8664	CLASH	23622248
hsa-let-7b-5p	MIMAT0000063	GBF1	8729	CLASH	23622248
hsa-let-7b-5p	MIMAT0000063	RNMT	8731	Proteomics	18668040
hsa-let-7b-5p	MIMAT0000063	TNFSF12	8742	CLASH	23622248
hsa-let-7b-5p	MIMAT0000063	TNFSF9	8744	PAR-CLIP	23592263|26701625
hsa-let-7b-5p	MIMAT0000063	SNAP23	8773	Proteomics//pSILAC	18668040
hsa-let-7b-5p	MIMAT0000063	RIOK3	8780	CLASH	23622248
hsa-let-7b-5p	MIMAT0000063	TNFRSF10B	8795	qRT-PCR//Western blot	24120475
hsa-let-7b-5p	MIMAT0000063	PEX11B	8799	PAR-CLIP//Proteomics	18668040|21572407
hsa-let-7b-5p	MIMAT0000063	TRIM24	8805	CLASH//Proteomics	18668040|23622248
hsa-let-7b-5p	MIMAT0000063	CCNK	8812	Proteomics	18668040
hsa-let-7b-5p	MIMAT0000063	SLC5A6	8884	PAR-CLIP	24398324|21572407
hsa-let-7b-5p	MIMAT0000063	DDX18	8886	CLASH	23622248
hsa-let-7b-5p	MIMAT0000063	EIF2B3	8891	Proteomics	18668040
hsa-let-7b-5p	MIMAT0000063	CCNA1	8900	Reporter assay	18379589

hsa-let-7b-5p	MIMAT0000063	MBD2	8932	PAR-CLIP	24398324|23446348|21572407|20371350
hsa-let-7b-5p	MIMAT0000063	WASF1	8936	CLASH	23622248
hsa-let-7b-5p	MIMAT0000063	WASL	8976	CLASH//PAR-CLIP	23622248|22012620|21572407|20371350
hsa-let-7b-5p	MIMAT0000063	BAZ1B	9031	CLASH//Proteomics	18668040|23622248
hsa-let-7b-5p	MIMAT0000063	UBA3	9039	Proteomics	18668040
hsa-let-7b-5p	MIMAT0000063	UBE2M	9040	Proteomics	18668040
hsa-let-7b-5p	MIMAT0000063	MAP7	9053	Proteomics	18668040
hsa-let-7b-5p	MIMAT0000063	PAPSS1	9061	Proteomics	18668040
hsa-let-7b-5p	MIMAT0000063	CLDN12	9069	PAR-CLIP	24398324|23446348|20371350|27292025
hsa-let-7b-5p	MIMAT0000063	USP14	9097	CLASH	23622248
hsa-let-7b-5p	MIMAT0000063	USP10	9100	Proteomics	18668040
hsa-let-7b-5p	MIMAT0000063	CCNB2	9133	CLASH	23622248
hsa-let-7b-5p	MIMAT0000063	ATG12	9140	PAR-CLIP	23592263
hsa-let-7b-5p	MIMAT0000063	SYNGR2	9144	CLASH//Proteomics	18668040|23622248
hsa-let-7b-5p	MIMAT0000063	HGS	9146	CLASH	23622248
hsa-let-7b-5p	MIMAT0000063	DDX21	9188	Proteomics	18668040
hsa-let-7b-5p	MIMAT0000063	DEDD	9191	CLASH	23622248
hsa-let-7b-5p	MIMAT0000063	AURKB	9212	Proteomics//pSILAC	18668040
hsa-let-7b-5p	MIMAT0000063	TIAF1	9220	PAR-CLIP	26701625
hsa-let-7b-5p	MIMAT0000063	NOLC1	9221	Proteomics	18668040
hsa-let-7b-5p	MIMAT0000063	PTTG1	9232	CLASH	23622248
hsa-let-7b-5p	MIMAT0000063	TBRG4	9238	Proteomics	18668040
hsa-let-7b-5p	MIMAT0000063	MED14	9282	CLASH	23622248
hsa-let-7b-5p	MIMAT0000063	ADGRG1	9289	Proteomics//pSILAC	18668040
hsa-let-7b-5p	MIMAT0000063	ATP6V1F	9296	PAR-CLIP//Proteomics//pSILAC	18668040|23592263|24398324|26701625|27292025
hsa-let-7b-5p	MIMAT0000063	COPS2	9318	Proteomics	18668040
hsa-let-7b-5p	MIMAT0000063	TRIP12	9320	Proteomics	18668040
hsa-let-7b-5p	MIMAT0000063	GTF3C4	9329	Proteomics	18668040
hsa-let-7b-5p	MIMAT0000063	VAMP3	9341	Proteomics	18668040
hsa-let-7b-5p	MIMAT0000063	SLC9A3R1	9368	Proteomics	18668040
hsa-let-7b-5p	MIMAT0000063	CIAO1	9391	Proteomics//pSILAC	18668040
hsa-let-7b-5p	MIMAT0000063	FADS2	9415	CLASH//Proteomics//pSILAC	18668040|23622248
hsa-let-7b-5p	MIMAT0000063	HAND1	9421	CLASH//PAR-CLIP	23622248|21572407

hsa-let-7b-5p	MIMAT0000063	ZNF264	9422	PAR-CLIP	21572407
hsa-let-7b-5p	MIMAT0000063	QKI	9444	CLASH	23622248
hsa-let-7b-5p	MIMAT0000063	GGPS1	9453	CLASH	23622248
hsa-let-7b-5p	MIMAT0000063	PCYT1B	9468	Proteomics	18668040
hsa-let-7b-5p	MIMAT0000063	ONECUT2	9480	PAR-CLIP	23446348
hsa-let-7b-5p	MIMAT0000063	FXR2	9513	CLASH	23622248
hsa-let-7b-5p	MIMAT0000063	EEF1E1	9521	Proteomics	18668040
hsa-let-7b-5p	MIMAT0000063	SCAMP1	9522	Proteomics	18668040
hsa-let-7b-5p	MIMAT0000063	BAG5	9529	Proteomics	18668040
hsa-let-7b-5p	MIMAT0000063	ATP6V1G1	9550	PAR-CLIP	22100165|22291592|23446348
hsa-let-7b-5p	MIMAT0000063	SOX13	9580	CLASH	23622248
hsa-let-7b-5p	MIMAT0000063	ENTPD4	9583	CLASH	23622248
hsa-let-7b-5p	MIMAT0000063	NCOR1	9611	CLASH	23622248
hsa-let-7b-5p	MIMAT0000063	NUP155	9631	CLASH//PAR-CLIP//Proteomics	18668040|23622248|23446348|21572407|20371350
hsa-let-7b-5p	MIMAT0000063	CLCA2	9635	CLASH	23622248
hsa-let-7b-5p	MIMAT0000063	SH3PXD2A	9644	CLASH	23622248
hsa-let-7b-5p	MIMAT0000063	CTR9	9646	CLASH	23622248
hsa-let-7b-5p	MIMAT0000063	HS2ST1	9653	Proteomics	18668040
hsa-let-7b-5p	MIMAT0000063	CEP135	9662	PAR-CLIP	24398324
hsa-let-7b-5p	MIMAT0000063	KDM4A	9682	CLASH	23622248
hsa-let-7b-5p	MIMAT0000063	CLINT1	9685	CLASH	23622248
hsa-let-7b-5p	MIMAT0000063	BZW1	9689	PAR-CLIP	23592263|20371350|26701625
hsa-let-7b-5p	MIMAT0000063	KIAA0391	9692	PAR-CLIP	21572407
hsa-let-7b-5p	MIMAT0000063	PUM1	9698	CLASH//Proteomics	18668040|23622248
hsa-let-7b-5p	MIMAT0000063	ESPL1	9700	PAR-CLIP	23446348|21572407
hsa-let-7b-5p	MIMAT0000063	HERPUD1	9709	HITS-CLIP//PAR-CLIP	23592263|23706177
hsa-let-7b-5p	MIMAT0000063	EIF4A3	9775	HITS-CLIP	23706177
hsa-let-7b-5p	MIMAT0000063	SNX17	9784	PAR-CLIP	22291592
hsa-let-7b-5p	MIMAT0000063	SCRN1	9805	Proteomics	18668040
hsa-let-7b-5p	MIMAT0000063	IP6K1	9807	CLASH	23622248
hsa-let-7b-5p	MIMAT0000063	RNF40	9810	Proteomics	18668040
hsa-let-7b-5p	MIMAT0000063	KIAA0141	9812	CLASH	23622248
hsa-let-7b-5p	MIMAT0000063	EFCAB14	9813	CLASH	23622248
hsa-let-7b-5p	MIMAT0000063	TSC22D2	9819	PAR-CLIP	23592263|24398324
hsa-let-7b-5p	MIMAT0000063	AREL1	9870	PAR-CLIP	23592263
hsa-let-7b-5p	MIMAT0000063	ZC3H11A	9877	Proteomics	18668040

hsa-let-7b-5p	MIMAT0000063	POM121	9883	CLASH//Proteomics//pSILAC	18668040|23622248
hsa-let-7b-5p	MIMAT0000063	SMG7	9887	CLASH	23622248
hsa-let-7b-5p	MIMAT0000063	UBAP2L	9898	CLASH//Proteomics	18668040|23622248
hsa-let-7b-5p	MIMAT0000063	RBM19	9904	Proteomics//pSILAC	18668040
hsa-let-7b-5p	MIMAT0000063	DENND4B	9909	CLASH	23622248
hsa-let-7b-5p	MIMAT0000063	NCAPD2	9918	CLASH	23622248
hsa-let-7b-5p	MIMAT0000063	SEC16A	9919	Proteomics	18668040
hsa-let-7b-5p	MIMAT0000063	ZBTB5	9925	PAR-CLIP	23592263|24398324|21572407|20371350
hsa-let-7b-5p	MIMAT0000063	LPGAT1	9926	Proteomics	18668040
hsa-let-7b-5p	MIMAT0000063	USP15	9958	Proteomics	18668040
hsa-let-7b-5p	MIMAT0000063	MED13	9969	CLASH	23622248
hsa-let-7b-5p	MIMAT0000063	NUP153	9972	CLASH	23622248
hsa-let-7b-5p	MIMAT0000063	HNRNPDL	9987	qRT-PCR	17942906
hsa-let-7b-5p	MIMAT0000063	CHAF1A	10036	Proteomics	18668040
hsa-let-7b-5p	MIMAT0000063	SCAMP3	10067	qRT-PCR	17942906
hsa-let-7b-5p	MIMAT0000063	HUWE1	10075	CLASH	23622248
hsa-let-7b-5p	MIMAT0000063	TSPAN3	10099	CLASH	23622248
hsa-let-7b-5p	MIMAT0000063	NUBP2	10101	Proteomics	18668040
hsa-let-7b-5p	MIMAT0000063	RBM12	10137	CLASH	23622248
hsa-let-7b-5p	MIMAT0000063	TRIM28	10155	CLASH	23622248
hsa-let-7b-5p	MIMAT0000063	FARP1	10160	Microarray	17699775
hsa-let-7b-5p	MIMAT0000063	LPCAT3	10162	Proteomics	18668040
hsa-let-7b-5p	MIMAT0000063	SLC25A13	10165	CLASH//Proteomics//pSILAC	18668040|23622248
hsa-let-7b-5p	MIMAT0000063	ALG3	10195	Proteomics//pSILAC	18668040
hsa-let-7b-5p	MIMAT0000063	PSME3	10197	CLASH//Proteomics	18668040|23622248
hsa-let-7b-5p	MIMAT0000063	NME6	10201	Proteomics	18668040
hsa-let-7b-5p	MIMAT0000063	GDF11	10220	CLASH	23622248
hsa-let-7b-5p	MIMAT0000063	ZNF443	10224	HITS-CLIP	23313552
hsa-let-7b-5p	MIMAT0000063	DCAF7	10238	CLASH	23622248
hsa-let-7b-5p	MIMAT0000063	CALCOCO2	10241	Proteomics//pSILAC	18668040
hsa-let-7b-5p	MIMAT0000063	GPHN	10243	CLASH	23622248
hsa-let-7b-5p	MIMAT0000063	AKAP8	10270	PAR-CLIP//Proteomics//pSILAC	18668040|21572407|20371350

hsa-let-7b-5p	MIMAT0000063	SIGMAR1	10280	Microarray//Proteomics//pSILAC	17699775|18668040
hsa-let-7b-5p	MIMAT0000063	MARCH6	10299	CLASH	23622248
hsa-let-7b-5p	MIMAT0000063	AKR1A1	10327	Proteomics	18668040
hsa-let-7b-5p	MIMAT0000063	PCGF3	10336	CLASH//PAR-CLIP	23622248|23592263|23446348|20371350
hsa-let-7b-5p	MIMAT0000063	WARS2	10352	Proteomics	18668040
hsa-let-7b-5p	MIMAT0000063	TUBA1B	10376	CLASH	23622248
hsa-let-7b-5p	MIMAT0000063	TUBB4A	10382	PAR-CLIP	26701625
hsa-let-7b-5p	MIMAT0000063	SCML2	10389	CLASH	23622248
hsa-let-7b-5p	MIMAT0000063	DLC1	10395	Microarray	17699775
hsa-let-7b-5p	MIMAT0000063	NDRG1	10397	CLASH	23622248
hsa-let-7b-5p	MIMAT0000063	NSA2	10412	Proteomics	18668040
hsa-let-7b-5p	MIMAT0000063	YAP1	10413	Microarray	17699775
hsa-let-7b-5p	MIMAT0000063	CD2BP2	10421	CLASH	23622248
hsa-let-7b-5p	MIMAT0000063	CDIPT	10423	Proteomics//pSILAC	18668040
hsa-let-7b-5p	MIMAT0000063	TUBGCP3	10426	CLASH	23622248
hsa-let-7b-5p	MIMAT0000063	ZER1	10444	CLASH	23622248
hsa-let-7b-5p	MIMAT0000063	SEC23B	10483	Proteomics	18668040
hsa-let-7b-5p	MIMAT0000063	LRRC41	10489	CLASH	23622248
hsa-let-7b-5p	MIMAT0000063	UNC13B	10497	CLASH	23622248
hsa-let-7b-5p	MIMAT0000063	DDX17	10521	CLASH	23622248
hsa-let-7b-5p	MIMAT0000063	IPO8	10526	Proteomics	18668040
hsa-let-7b-5p	MIMAT0000063	IPO7	10527	CLASH	23622248
hsa-let-7b-5p	MIMAT0000063	RPP38	10557	Proteomics//pSILAC	18668040
hsa-let-7b-5p	MIMAT0000063	PRPF8	10594	CLASH	23622248
hsa-let-7b-5p	MIMAT0000063	PDLIM5	10611	PAR-CLIP	26701625
hsa-let-7b-5p	MIMAT0000063	TGOLN2	10618	PAR-CLIP	20371350
hsa-let-7b-5p	MIMAT0000063	ARID3B	10620	CLASH//PAR-CLIP	23622248|23446348|21572407|20371350
hsa-let-7b-5p	MIMAT0000063	POLR3G	10622	CLASH	23622248
hsa-let-7b-5p	MIMAT0000063	LEFTY1	10637	PAR-CLIP	22012620
hsa-let-7b-5p	MIMAT0000063	IGF2BP1	10642	Immunoblot//Immunofluorescence//Luciferase reporter assay//PAR-CLIP//Proteomics//pSILAC//qRT-PCR//Western blot	18668040|21252116|21572407|23824794
hsa-let-7b-5p	MIMAT0000063	IGF2BP3	10643	PAR-CLIP//Proteomics	18668040|21572407|20371350

hsa-let-7b-5p	MIMAT0000063	IGF2BP2	10644	Immunohistochemistry//Luciferase reporter assay//Proteomics//pSILAC//QRTPCR//Western blot	18668040|23482325
hsa-let-7b-5p	MIMAT0000063	CELF1	10658	PAR-CLIP	24398324
hsa-let-7b-5p	MIMAT0000063	CTCF	10664	CLASH	23622248
hsa-let-7b-5p	MIMAT0000063	SLC12A7	10723	PAR-CLIP	22291592
hsa-let-7b-5p	MIMAT0000063	RAI1	10743	CLASH	23622248
hsa-let-7b-5p	MIMAT0000063	KIF1C	10749	CLASH	23622248
hsa-let-7b-5p	MIMAT0000063	AHCYL1	10768	CLASH	23622248
hsa-let-7b-5p	MIMAT0000063	ARPP19	10776	CLASH	23622248
hsa-let-7b-5p	MIMAT0000063	WDR4	10785	Proteomics	18668040
hsa-let-7b-5p	MIMAT0000063	ZNF460	10794	PAR-CLIP	21572407|20371350
hsa-let-7b-5p	MIMAT0000063	TUBGCP2	10844	Proteomics	18668040
hsa-let-7b-5p	MIMAT0000063	CLPX	10845	Proteomics	18668040
hsa-let-7b-5p	MIMAT0000063	SRCAP	10847	CLASH	23622248
hsa-let-7b-5p	MIMAT0000063	PGRMC1	10857	PAR-CLIP//Proteomics//pSILAC	18668040|21572407
hsa-let-7b-5p	MIMAT0000063	WDR3	10885	CLASH	23622248
hsa-let-7b-5p	MIMAT0000063	RAB10	10890	CLASH	23622248
hsa-let-7b-5p	MIMAT0000063	PPARGC1A	10891	CLASH	23622248
hsa-let-7b-5p	MIMAT0000063	TXNL4A	10907	CLASH	23622248
hsa-let-7b-5p	MIMAT0000063	PAPOLA	10914	CLASH	23622248
hsa-let-7b-5p	MIMAT0000063	POP1	10940	Proteomics	18668040
hsa-let-7b-5p	MIMAT0000063	KDELR1	10945	CLASH	23622248
hsa-let-7b-5p	MIMAT0000063	LMAN2	10960	CLASH	23622248
hsa-let-7b-5p	MIMAT0000063	STIP1	10963	CLASH	23622248
hsa-let-7b-5p	MIMAT0000063	ASCC3	10973	Proteomics	18668040
hsa-let-7b-5p	MIMAT0000063	GCN1	10985	CLASH	23622248
hsa-let-7b-5p	MIMAT0000063	SLC27A2	11001	Proteomics	18668040
hsa-let-7b-5p	MIMAT0000063	ADRM1	11047	CLASH	23622248
hsa-let-7b-5p	MIMAT0000063	TMEM115	11070	CLASH	23622248
hsa-let-7b-5p	MIMAT0000063	HNRNPUL1	11100	CLASH	23622248
hsa-let-7b-5p	MIMAT0000063	ATE1	11101	CLASH	23622248
hsa-let-7b-5p	MIMAT0000063	PRDM4	11108	CLASH	23622248
hsa-let-7b-5p	MIMAT0000063	LSM6	11157	Proteomics	18668040

hsa-let-7b-5p	MIMAT0000063	RABL2B	11158	PAR-CLIP	23592263|26701625
hsa-let-7b-5p	MIMAT0000063	RABL2A	11159	PAR-CLIP	23592263|26701625
hsa-let-7b-5p	MIMAT0000063	FSTL1	11167	CLASH	23622248
hsa-let-7b-5p	MIMAT0000063	BAZ2A	11176	CLASH	23622248
hsa-let-7b-5p	MIMAT0000063	BAZ1A	11177	Proteomics	18668040
hsa-let-7b-5p	MIMAT0000063	ABCB8	11194	CLASH	23622248
hsa-let-7b-5p	MIMAT0000063	SUPT16H	11198	CLASH	23622248
hsa-let-7b-5p	MIMAT0000063	DDX20	11218	CLASH	23622248
hsa-let-7b-5p	MIMAT0000063	PRAF2	11230	CLASH	23622248
hsa-let-7b-5p	MIMAT0000063	PDCD10	11235	Proteomics	18668040
hsa-let-7b-5p	MIMAT0000063	PMF1	11243	Proteomics	18668040
hsa-let-7b-5p	MIMAT0000063	DUSP12	11266	Proteomics//pSILAC	18668040
hsa-let-7b-5p	MIMAT0000063	ATXN2L	11273	CLASH	23622248
hsa-let-7b-5p	MIMAT0000063	STK38	11329	CLASH	23622248
hsa-let-7b-5p	MIMAT0000063	IKZF3	22806	HITS-CLIP//PAR-CLIP	23446348|23706177
hsa-let-7b-5p	MIMAT0000063	COPG1	22820	Proteomics	18668040
hsa-let-7b-5p	MIMAT0000063	DNAJC8	22826	CLASH	23622248
hsa-let-7b-5p	MIMAT0000063	SCAF8	22828	CLASH	23622248
hsa-let-7b-5p	MIMAT0000063	ZNF652	22834	CLASH	23622248
hsa-let-7b-5p	MIMAT0000063	RNF44	22838	PAR-CLIP	24398324|20371350|27292025
hsa-let-7b-5p	MIMAT0000063	ZNF507	22847	CLASH	23622248
hsa-let-7b-5p	MIMAT0000063	CPEB3	22849	Luciferase reporter assay//Microarray//qRT-PCR	22995917
hsa-let-7b-5p	MIMAT0000063	FNDC3A	22862	PAR-CLIP//Proteomics//pSILAC	18668040|23592263
hsa-let-7b-5p	MIMAT0000063	DZIP1	22873	Microarray	17699775
hsa-let-7b-5p	MIMAT0000063	BAHD1	22893	CLASH	23622248
hsa-let-7b-5p	MIMAT0000063	RPIA	22934	Luciferase reporter assay//Reporter assay	15131085
hsa-let-7b-5p	MIMAT0000063	PDCD11	22984	CLASH//Proteomics	18668040|23622248
hsa-let-7b-5p	MIMAT0000063	CNOT1	23019	CLASH	23622248
hsa-let-7b-5p	MIMAT0000063	RBM34	23029	Proteomics	18668040
hsa-let-7b-5p	MIMAT0000063	XPO7	23039	Proteomics	18668040
hsa-let-7b-5p	MIMAT0000063	SMG1	23049	CLASH	23622248
hsa-let-7b-5p	MIMAT0000063	ZNF609	23060	PAR-CLIP	26701625
hsa-let-7b-5p	MIMAT0000063	RRP1B	23076	Proteomics//pSILAC	18668040
hsa-let-7b-5p	MIMAT0000063	VWA8	23078	Proteomics	18668040

hsa-let-7b-5p	MIMAT0000063	PPRC1	23082	CLASH	23622248
hsa-let-7b-5p	MIMAT0000063	ERC1	23085	Proteomics//pSILAC	18668040
hsa-let-7b-5p	MIMAT0000063	PEG10	23089	PAR-CLIP	20371350
hsa-let-7b-5p	MIMAT0000063	ARHGAP 26	23092	CLASH	23622248
hsa-let-7b-5p	MIMAT0000063	MCF2L2	23101	HITS-CLIP//PAR-CLIP	21572407|20371350|23706177
hsa-let-7b-5p	MIMAT0000063	TNRC6B	23112	CLASH	23622248
hsa-let-7b-5p	MIMAT0000063	TAB2	23118	CLASH//Microarray	17699775|23622248
hsa-let-7b-5p	MIMAT0000063	PLXND1	23129	PAR-CLIP	23592263
hsa-let-7b-5p	MIMAT0000063	EPB41L3	23136	CLASH	23622248
hsa-let-7b-5p	MIMAT0000063	GGA3	23163	PAR-CLIP	24398324
hsa-let-7b-5p	MIMAT0000063	TTLL12	23170	Proteomics	18668040
hsa-let-7b-5p	MIMAT0000063	ATG4B	23192	Proteomics	18668040
hsa-let-7b-5p	MIMAT0000063	PMPCA	23203	PAR-CLIP	21572407|20371350
hsa-let-7b-5p	MIMAT0000063	ARL6IP1	23204	CLASH	23622248
hsa-let-7b-5p	MIMAT0000063	SYNE2	23224	CLASH	23622248
hsa-let-7b-5p	MIMAT0000063	PDS5A	23244	CLASH	23622248
hsa-let-7b-5p	MIMAT0000063	ADGRL2	23266	CLASH	23622248
hsa-let-7b-5p	MIMAT0000063	DNMBP	23268	CLASH	23622248
hsa-let-7b-5p	MIMAT0000063	CLUH	23277	CLASH//Proteomics	18668040|23622248
hsa-let-7b-5p	MIMAT0000063	ICOSLG	23308	PAR-CLIP	23592263
hsa-let-7b-5p	MIMAT0000063	KIAA093 0	23313	PAR-CLIP	23592263
hsa-let-7b-5p	MIMAT0000063	ZCCHC11	23318	CLASH	23622248
hsa-let-7b-5p	MIMAT0000063	USP22	23326	CLASH	23622248
hsa-let-7b-5p	MIMAT0000063	VPS39	23339	Proteomics//pSILAC	18668040
hsa-let-7b-5p	MIMAT0000063	SYNE1	23345	Proteomics	18668040
hsa-let-7b-5p	MIMAT0000063	ZNF629	23361	CLASH	23622248
hsa-let-7b-5p	MIMAT0000063	PSD3	23362	CLASH	23622248
hsa-let-7b-5p	MIMAT0000063	LARP1	23367	CLASH	23622248
hsa-let-7b-5p	MIMAT0000063	RRP8	23378	Proteomics//pSILAC	18668040
hsa-let-7b-5p	MIMAT0000063	AHCYL2	23382	PAR-CLIP	23592263
hsa-let-7b-5p	MIMAT0000063	ADNP	23394	CLASH	23622248
hsa-let-7b-5p	MIMAT0000063	DICER1	23405	Immunoblot	18812516
hsa-let-7b-5p	MIMAT0000063	SF3B1	23451	CLASH	23622248
hsa-let-7b-5p	MIMAT0000063	ABCB10	23456	Proteomics	18668040
hsa-let-7b-5p	MIMAT0000063	CBX6	23466	CLASH	23622248
hsa-let-7b-5p	MIMAT0000063	CBX5	23468	CLASH//PAR-CLIP	23622248|21572407
hsa-let-7b-5p	MIMAT0000063	SEC11A	23478	Proteomics	18668040

hsa-let-7b-5p	MIMAT0000063	RBFOX2	23543	CLASH//PAR-CLIP//Proteomics	18668040|23622248|24398324|23446348|22012620|21572407|20371350
hsa-let-7b-5p	MIMAT0000063	CARHSP1	23589	Proteomics//pSILAC	18668040
hsa-let-7b-5p	MIMAT0000063	ACOT9	23597	PAR-CLIP	23592263|23446348
hsa-let-7b-5p	MIMAT0000063	MKRN2	23609	Proteomics	18668040
hsa-let-7b-5p	MIMAT0000063	KPNA6	23633	CLASH	23622248
hsa-let-7b-5p	MIMAT0000063	PLD3	23646	PAR-CLIP	26701625
hsa-let-7b-5p	MIMAT0000063	ARFIP2	23647	Proteomics	18668040
hsa-let-7b-5p	MIMAT0000063	PLXNB2	23654	CLASH	23622248
hsa-let-7b-5p	MIMAT0000063	TMEM2	23670	Proteomics//pSILAC	18668040
hsa-let-7b-5p	MIMAT0000063	RAB38	23682	Proteomics	18668040
hsa-let-7b-5p	MIMAT0000063	IFIT5	24138	Proteomics//pSILAC	18668040
hsa-let-7b-5p	MIMAT0000063	RAB3GAP2	25782	Proteomics	18668040
hsa-let-7b-5p	MIMAT0000063	CIZ1	25792	CLASH	23622248
hsa-let-7b-5p	MIMAT0000063	ARIH1	25820	PAR-CLIP	21572407
hsa-let-7b-5p	MIMAT0000063	NIPBL	25836	Proteomics	18668040
hsa-let-7b-5p	MIMAT0000063	YIPF3	25844	CLASH	23622248
hsa-let-7b-5p	MIMAT0000063	POLR1A	25885	Proteomics	18668040
hsa-let-7b-5p	MIMAT0000063	INTS7	25896	HITS-CLIP//Proteomics	18668040|23706177
hsa-let-7b-5p	MIMAT0000063	AHCTF1	25909	Proteomics	18668040
hsa-let-7b-5p	MIMAT0000063	GEMIN5	25929	Proteomics	18668040
hsa-let-7b-5p	MIMAT0000063	PTPN23	25930	CLASH	23622248
hsa-let-7b-5p	MIMAT0000063	VIRMA	25962	CLASH	23622248
hsa-let-7b-5p	MIMAT0000063	MMACHC	25974	CLASH	23622248
hsa-let-7b-5p	MIMAT0000063	RPAP1	26015	CLASH	23622248
hsa-let-7b-5p	MIMAT0000063	LRIG1	26018	Luciferase reporter assay//Microarray//qRT-PCR	22995917
hsa-let-7b-5p	MIMAT0000063	PTCD1	26024	Proteomics	18668040
hsa-let-7b-5p	MIMAT0000063	IPCEF1	26034	CLASH	23622248
hsa-let-7b-5p	MIMAT0000063	PPP1R16B	26051	CLASH	23622248
hsa-let-7b-5p	MIMAT0000063	DNM3	26052	Proteomics	18668040
hsa-let-7b-5p	MIMAT0000063	ANKRD17	26057	CLASH	23622248
hsa-let-7b-5p	MIMAT0000063	APPL1	26060	CLASH	23622248
hsa-let-7b-5p	MIMAT0000063	CHTOP	26097	HITS-CLIP	23313552
hsa-let-7b-5p	MIMAT0000063	SZRD1	26099	CLASH	23622248
hsa-let-7b-5p	MIMAT0000063	GAPVD1	26130	Proteomics	18668040

hsa-let-7b-5p	MIMAT0000063	SERBP1	26135	CLASH	23622248
hsa-let-7b-5p	MIMAT0000063	TES	26136	Proteomics	18668040
hsa-let-7b-5p	MIMAT0000063	INTS1	26173	CLASH	23622248
hsa-let-7b-5p	MIMAT0000063	FBXW2	26190	CLASH//PAR-CLIP	23622248|23446348|20371350
hsa-let-7b-5p	MIMAT0000063	MYCBP	26292	Proteomics	18668040
hsa-let-7b-5p	MIMAT0000063	CNNM3	26505	CLASH	23622248
hsa-let-7b-5p	MIMAT0000063	TIMM9	26520	Proteomics	18668040
hsa-let-7b-5p	MIMAT0000063	AGO1	26523	CLASH//Immunohistochemistry//Immunoprecipitaion//Luciferase reporter assay//Northern blot//qRT-PCR//Western blot	23622248|23426184
hsa-let-7b-5p	MIMAT0000063	AATF	26574	CLASH	23622248
hsa-let-7b-5p	MIMAT0000063	CKAP2	26586	CLASH	23622248
hsa-let-7b-5p	MIMAT0000063	RANBP6	26953	Proteomics	18668040
hsa-let-7b-5p	MIMAT0000063	AP3M1	26985	CLASH	23622248
hsa-let-7b-5p	MIMAT0000063	PABPC1	26986	CLASH	23622248
hsa-let-7b-5p	MIMAT0000063	TRUB2	26995	Proteomics	18668040
hsa-let-7b-5p	MIMAT0000063	NPTN	27020	CLASH	23622248
hsa-let-7b-5p	MIMAT0000063	VPS41	27072	CLASH	23622248
hsa-let-7b-5p	MIMAT0000063	EIF2AK1	27102	CLASH	23622248
hsa-let-7b-5p	MIMAT0000063	AFF4	27125	CLASH	23622248
hsa-let-7b-5p	MIMAT0000063	PALD1	27143	CLASH	23622248
hsa-let-7b-5p	MIMAT0000063	BRPF3	27154	CLASH	23622248
hsa-let-7b-5p	MIMAT0000063	AGO2	27161	CLASH	23622248
hsa-let-7b-5p	MIMAT0000063	SALL3	27164	PAR-CLIP	21572407
hsa-let-7b-5p	MIMAT0000063	DISC1	27185	PAR-CLIP	23592263|26701625|27292025
hsa-let-7b-5p	MIMAT0000063	CHMP2A	27243	Proteomics//pSILAC	18668040
hsa-let-7b-5p	MIMAT0000063	RNF115	27246	CLASH	23622248
hsa-let-7b-5p	MIMAT0000063	NFU1	27247	CLASH	23622248
hsa-let-7b-5p	MIMAT0000063	TOX3	27324	CLASH	23622248
hsa-let-7b-5p	MIMAT0000063	PRPF19	27339	CLASH	23622248
hsa-let-7b-5p	MIMAT0000063	RRP7A	27341	Proteomics	18668040
hsa-let-7b-5p	MIMAT0000063	POLL	27343	PAR-CLIP	26701625
hsa-let-7b-5p	MIMAT0000063	MCAT	27349	Proteomics	18668040
hsa-let-7b-5p	MIMAT0000063	MAT2B	27430	Proteomics	18668040
hsa-let-7b-5p	MIMAT0000063	OSTM1	28962	CLASH	23622248
hsa-let-7b-5p	MIMAT0000063	BZW2	28969	Proteomics	18668040

hsa-let-7b-5p	MIMAT0000063	C19orf53	28974	PAR-CLIP	23592263|26701625
hsa-let-7b-5p	MIMAT0000063	DBNL	28988	CLASH	23622248
hsa-let-7b-5p	MIMAT0000063	THYN1	29087	PAR-CLIP	20371350
hsa-let-7b-5p	MIMAT0000063	COMMD9	29099	Proteomics//pSILAC	18668040
hsa-let-7b-5p	MIMAT0000063	UHRF1	29128	Proteomics//pSILAC	18668040
hsa-let-7b-5p	MIMAT0000063	ABT1	29777	HITS-CLIP//PAR-CLIP	23592263|24398324|21572407|23313552|27292025
hsa-let-7b-5p	MIMAT0000063	PARVB	29780	Proteomics	18668040
hsa-let-7b-5p	MIMAT0000063	CPSF1	29894	Proteomics	18668040
hsa-let-7b-5p	MIMAT0000063	SNX12	29934	CLASH	23622248
hsa-let-7b-5p	MIMAT0000063	SLC25A24	29957	Proteomics//pSILAC	18668040
hsa-let-7b-5p	MIMAT0000063	NOP53	29997	Proteomics	18668040
hsa-let-7b-5p	MIMAT0000063	ERO1A	30001	PAR-CLIP//Proteomics	18668040|26701625
hsa-let-7b-5p	MIMAT0000063	SOCS7	30837	PAR-CLIP	26701625
hsa-let-7b-5p	MIMAT0000063	EHD4	30844	Proteomics	18668040
hsa-let-7b-5p	MIMAT0000063	TMED5	50999	HITS-CLIP//PAR-CLIP//Proteomics	18668040|23592263|24906430
hsa-let-7b-5p	MIMAT0000063	TRNT1	51095	Proteomics	18668040
hsa-let-7b-5p	MIMAT0000063	SH3GLB1	51100	Proteomics	18668040
hsa-let-7b-5p	MIMAT0000063	LACTB2	51110	CLASH	23622248
hsa-let-7b-5p	MIMAT0000063	NAA20	51126	CLASH//PAR-CLIP	23622248|23592263
hsa-let-7b-5p	MIMAT0000063	RNFT1	51136	PAR-CLIP	24398324
hsa-let-7b-5p	MIMAT0000063	VPS28	51160	CLASH	23622248
hsa-let-7b-5p	MIMAT0000063	PLEKHO1	51177	PAR-CLIP	22291592|20371350
hsa-let-7b-5p	MIMAT0000063	IPO11	51194	CLASH	23622248
hsa-let-7b-5p	MIMAT0000063	CPA4	51200	HITS-CLIP	23706177
hsa-let-7b-5p	MIMAT0000063	NUSAP1	51203	CLASH	23622248
hsa-let-7b-5p	MIMAT0000063	HGH1	51236	pSILAC	18668040
hsa-let-7b-5p	MIMAT0000063	MRPL37	51253	CLASH	23622248
hsa-let-7b-5p	MIMAT0000063	C1RL	51279	PAR-CLIP	23592263
hsa-let-7b-5p	MIMAT0000063	ERGIC2	51290	Proteomics	18668040
hsa-let-7b-5p	MIMAT0000063	THEM6	51337	PAR-CLIP	23592263|24398324
hsa-let-7b-5p	MIMAT0000063	RWDD1	51389	HITS-CLIP	23824327
hsa-let-7b-5p	MIMAT0000063	DDX41	51428	Proteomics	18668040
hsa-let-7b-5p	MIMAT0000063	SFMBT1	51460	CLASH	23622248
hsa-let-7b-5p	MIMAT0000063	NCKIPSD	51517	HITS-CLIP//PAR-CLIP	23446348|23706177
hsa-let-7b-5p	MIMAT0000063	TRMO	51531	PAR-CLIP	23446348
hsa-let-7b-5p	MIMAT0000063	MTFP1	51537	Proteomics	18668040

hsa-let-7b-5p	MIMAT0000063	ZNF581	51545	CLASH	23622248
hsa-let-7b-5p	MIMAT0000063	FAM49B	51571	Proteomics	18668040
hsa-let-7b-5p	MIMAT0000063	GDE1	51573	Proteomics//pSILAC	18668040
hsa-let-7b-5p	MIMAT0000063	ERGIC3	51614	Proteomics	18668040
hsa-let-7b-5p	MIMAT0000063	TAF9B	51616	Proteomics//pSILAC	18668040
hsa-let-7b-5p	MIMAT0000063	MRPS33	51650	Proteomics//pSILAC	18668040
hsa-let-7b-5p	MIMAT0000063	CHMP3	51652	Proteomics	18668040
hsa-let-7b-5p	MIMAT0000063	WBP11	51729	CLASH	23622248
hsa-let-7b-5p	MIMAT0000063	FGFRL1	53834	CLASH	23622248
hsa-let-7b-5p	MIMAT0000063	CHRAC1	54108	Proteomics	18668040
hsa-let-7b-5p	MIMAT0000063	TERF2IP	54386	CLASH	23622248
hsa-let-7b-5p	MIMAT0000063	SLC38A2	54407	Proteomics	18668040
hsa-let-7b-5p	MIMAT0000063	XRN1	54464	Proteomics	18668040
hsa-let-7b-5p	MIMAT0000063	MIOS	54468	Proteomics	18668040
hsa-let-7b-5p	MIMAT0000063	MIEF1	54471	PAR-CLIP	23592263|24398324|21572407|20371350
hsa-let-7b-5p	MIMAT0000063	NLE1	54475	Proteomics	18668040
hsa-let-7b-5p	MIMAT0000063	CHPF2	54480	Proteomics//pSILAC	18668040
hsa-let-7b-5p	MIMAT0000063	FAM105A	54491	HITS-CLIP//PAR-CLIP//Proteomics//pSILAC	18668040|23592263|23446348|23706177
hsa-let-7b-5p	MIMAT0000063	MIER2	54531	CLASH	23622248
hsa-let-7b-5p	MIMAT0000063	DDX49	54555	Proteomics	18668040
hsa-let-7b-5p	MIMAT0000063	CCNJ	54619	Microarray	17699775
hsa-let-7b-5p	MIMAT0000063	ARL15	54622	Proteomics//pSILAC	18668040
hsa-let-7b-5p	MIMAT0000063	TBC1D13	54662	Proteomics	18668040
hsa-let-7b-5p	MIMAT0000063	WDR74	54663	Proteomics	18668040
hsa-let-7b-5p	MIMAT0000063	MANSC1	54682	CLASH	23622248
hsa-let-7b-5p	MIMAT0000063	RBFOX1	54715	Proteomics	18668040
hsa-let-7b-5p	MIMAT0000063	PPP1R12C	54776	CLASH	23622248
hsa-let-7b-5p	MIMAT0000063	NSMCE4A	54780	Proteomics	18668040
hsa-let-7b-5p	MIMAT0000063	HAUS6	54801	CLASH	23622248
hsa-let-7b-5p	MIMAT0000063	WDR55	54853	CLASH	23622248
hsa-let-7b-5p	MIMAT0000063	TOR4A	54863	CLASH	23622248
hsa-let-7b-5p	MIMAT0000063	GPATCH4	54865	Proteomics	18668040
hsa-let-7b-5p	MIMAT0000063	PIGG	54872	CLASH	23622248
hsa-let-7b-5p	MIMAT0000063	BCOR	54880	CLASH	23622248
hsa-let-7b-5p	MIMAT0000063	NSUN2	54888	CLASH	23622248

hsa-let-7b-5p	MIMAT0000063	NCAPG2	54892	Proteomics//pSILAC	18668040
hsa-let-7b-5p	MIMAT0000063	CDKAL1	54901	HITS-CLIP//Proteomics//pSILAC	18668040|23706177
hsa-let-7b-5p	MIMAT0000063	SEMA4C	54910	PAR-CLIP	24398324|23446348|20371350
hsa-let-7b-5p	MIMAT0000063	DNAAF5	54919	Proteomics	18668040
hsa-let-7b-5p	MIMAT0000063	DUSP23	54935	Proteomics//pSILAC	18668040
hsa-let-7b-5p	MIMAT0000063	DNAJC28	54943	HITS-CLIP	23313552
hsa-let-7b-5p	MIMAT0000063	C1orf27	54953	Proteomics//pSILAC	18668040
hsa-let-7b-5p	MIMAT0000063	PARP16	54956	PAR-CLIP	20371350
hsa-let-7b-5p	MIMAT0000063	INTS11	54973	Proteomics	18668040
hsa-let-7b-5p	MIMAT0000063	SLC35F6	54978	Proteomics//pSILAC	18668040
hsa-let-7b-5p	MIMAT0000063	PIH1D1	55011	Proteomics	18668040
hsa-let-7b-5p	MIMAT0000063	USP47	55031	PAR-CLIP	27292025
hsa-let-7b-5p	MIMAT0000063	PDPR	55066	Proteomics	18668040
hsa-let-7b-5p	MIMAT0000063	OXR1	55074	CLASH	23622248
hsa-let-7b-5p	MIMAT0000063	CCDC18 6	55088	CLASH	23622248
hsa-let-7b-5p	MIMAT0000063	GPATCH 1	55094	CLASH	23622248
hsa-let-7b-5p	MIMAT0000063	ARHGAP 17	55114	Proteomics	18668040
hsa-let-7b-5p	MIMAT0000063	FIGN	55137	HITS-CLIP//PAR-CLIP	23446348|21572407|20371350|2370617 7
hsa-let-7b-5p	MIMAT0000063	ANKZF1	55139	Proteomics	18668040
hsa-let-7b-5p	MIMAT0000063	CDCA8	55143	Proteomics	18668040
hsa-let-7b-5p	MIMAT0000063	SDAD1	55153	Proteomics	18668040
hsa-let-7b-5p	MIMAT0000063	TMEM33	55161	Proteomics	18668040
hsa-let-7b-5p	MIMAT0000063	KLHL11	55175	CLASH	23622248
hsa-let-7b-5p	MIMAT0000063	MRM3	55178	Proteomics	18668040
hsa-let-7b-5p	MIMAT0000063	ARL8B	55207	HITS-CLIP	23706177
hsa-let-7b-5p	MIMAT0000063	SETD5	55209	CLASH	23622248
hsa-let-7b-5p	MIMAT0000063	BBS7	55212	CLASH	23622248
hsa-let-7b-5p	MIMAT0000063	C11orf5 7	55216	PAR-CLIP	23592263|21572407
hsa-let-7b-5p	MIMAT0000063	LRRC20	55222	PAR-CLIP	23592263
hsa-let-7b-5p	MIMAT0000063	MOB1A	55233	Proteomics	18668040
hsa-let-7b-5p	MIMAT0000063	SLC38A7	55238	HITS-CLIP	23706177
hsa-let-7b-5p	MIMAT0000063	STEAP3	55240	CLASH	23622248
hsa-let-7b-5p	MIMAT0000063	QRSL1	55278	Proteomics	18668040
hsa-let-7b-5p	MIMAT0000063	TBC1D19	55296	PAR-CLIP	22291592
hsa-let-7b-5p	MIMAT0000063	SYNJ2BP	55333	PAR-CLIP	23592263|26701625

hsa-let-7b-5p	MIMAT0000063	WDR33	55339	Proteomics	18668040
hsa-let-7b-5p	MIMAT0000063	LSG1	55341	Proteomics	18668040
hsa-let-7b-5p	MIMAT0000063	TMEM63B	55362	CLASH	23622248
hsa-let-7b-5p	MIMAT0000063	LGR4	55366	Flow//Luciferase reporter assay//qRT-PCR//Western blot	27179410
hsa-let-7b-5p	MIMAT0000063	YOD1	55432	PAR-CLIP	23446348|21572407|20371350
hsa-let-7b-5p	MIMAT0000063	NDUFAF7	55471	Proteomics	18668040
hsa-let-7b-5p	MIMAT0000063	DHTKD1	55526	CLASH	23622248
hsa-let-7b-5p	MIMAT0000063	FOXRED1	55572	Proteomics	18668040
hsa-let-7b-5p	MIMAT0000063	CDV3	55573	PAR-CLIP	21572407
hsa-let-7b-5p	MIMAT0000063	SUPT20H	55578	CLASH	23622248
hsa-let-7b-5p	MIMAT0000063	KIF27	55582	PAR-CLIP	22100165
hsa-let-7b-5p	MIMAT0000063	UBE2Q1	55585	CLASH	23622248
hsa-let-7b-5p	MIMAT0000063	OTUB1	55611	CLASH	23622248
hsa-let-7b-5p	MIMAT0000063	TRMT1	55621	Proteomics//pSILAC	18668040
hsa-let-7b-5p	MIMAT0000063	LRRC40	55631	Proteomics	18668040
hsa-let-7b-5p	MIMAT0000063	CHD7	55636	CLASH	23622248
hsa-let-7b-5p	MIMAT0000063	BCAS4	55653	CLASH	23622248
hsa-let-7b-5p	MIMAT0000063	YEATS2	55689	CLASH	23622248
hsa-let-7b-5p	MIMAT0000063	RBM22	55696	CLASH	23622248
hsa-let-7b-5p	MIMAT0000063	IARS2	55699	CLASH	23622248
hsa-let-7b-5p	MIMAT0000063	MAP7D1	55700	Proteomics//pSILAC	18668040
hsa-let-7b-5p	MIMAT0000063	POLR3B	55703	Proteomics	18668040
hsa-let-7b-5p	MIMAT0000063	IPO9	55705	HITS-CLIP	23313552
hsa-let-7b-5p	MIMAT0000063	TENM3	55714	CLASH	23622248
hsa-let-7b-5p	MIMAT0000063	SLF2	55719	CLASH	23622248
hsa-let-7b-5p	MIMAT0000063	FAM222B	55731	PAR-CLIP	21572407
hsa-let-7b-5p	MIMAT0000063	DNAJC11	55735	Proteomics	18668040
hsa-let-7b-5p	MIMAT0000063	CNDP2	55748	Proteomics	18668040
hsa-let-7b-5p	MIMAT0000063	RIOK2	55781	Proteomics	18668040
hsa-let-7b-5p	MIMAT0000063	TXLNG	55787	PAR-CLIP	21572407
hsa-let-7b-5p	MIMAT0000063	DDX28	55794	Proteomics	18668040
hsa-let-7b-5p	MIMAT0000063	UTP6	55813	Proteomics	18668040
hsa-let-7b-5p	MIMAT0000063	DBNDD2	55861	CLASH	23622248
hsa-let-7b-5p	MIMAT0000063	ECHDC1	55862	PAR-CLIP	21572407
hsa-let-7b-5p	MIMAT0000063	NXT2	55916	Microarray	17699775
hsa-let-7b-5p	MIMAT0000063	YLPM1	56252	CLASH	23622248

hsa-let-7b-5p	MIMAT0000063	LRRC8A	56262	Proteomics	18668040
hsa-let-7b-5p	MIMAT0000063	KYAT3	56267	Proteomics//pSILAC	18668040
hsa-let-7b-5p	MIMAT0000063	DIABLO	56616	PAR-CLIP	23446348|20371350
hsa-let-7b-5p	MIMAT0000063	SAR1A	56681	HITS-CLIP	23706177
hsa-let-7b-5p	MIMAT0000063	ZC3HAV1	56829	CLASH	23622248
hsa-let-7b-5p	MIMAT0000063	RAD18	56852	PAR-CLIP	21572407
hsa-let-7b-5p	MIMAT0000063	DPYSL5	56896	CLASH	23622248
hsa-let-7b-5p	MIMAT0000063	C1GALT1	56913	CLASH	23622248
hsa-let-7b-5p	MIMAT0000063	SMARCAD1	56916	PAR-CLIP	24398324|20371350
hsa-let-7b-5p	MIMAT0000063	DHX33	56919	Proteomics	18668040
hsa-let-7b-5p	MIMAT0000063	C5orf15	56951	CLASH	23622248
hsa-let-7b-5p	MIMAT0000063	ATXN7L3	56970	PAR-CLIP	23592263
hsa-let-7b-5p	MIMAT0000063	YAE1D1	57002	PAR-CLIP	23592263
hsa-let-7b-5p	MIMAT0000063	PITHD1	57095	Proteomics	18668040
hsa-let-7b-5p	MIMAT0000063	C12orf4	57102	PAR-CLIP	23446348
hsa-let-7b-5p	MIMAT0000063	MRPL47	57129	Proteomics	18668040
hsa-let-7b-5p	MIMAT0000063	RALGAPB	57148	CLASH	23622248
hsa-let-7b-5p	MIMAT0000063	SPRYD7	57213	Proteomics	18668040
hsa-let-7b-5p	MIMAT0000063	NHSL1	57224	CLASH	23622248
hsa-let-7b-5p	MIMAT0000063	SCYL1	57410	Proteomics//pSILAC	18668040
hsa-let-7b-5p	MIMAT0000063	BIRC6	57448	qRT-PCR	17942906
hsa-let-7b-5p	MIMAT0000063	KIAA1143	57456	PAR-CLIP	26701625
hsa-let-7b-5p	MIMAT0000063	SCAF4	57466	Proteomics	18668040
hsa-let-7b-5p	MIMAT0000063	LRRC47	57470	Proteomics	18668040
hsa-let-7b-5p	MIMAT0000063	KIDINS220	57498	Proteomics	18668040
hsa-let-7b-5p	MIMAT0000063	MTUS1	57509	CLASH//PAR-CLIP	23622248|23592263|24398324|23446348|26701625
hsa-let-7b-5p	MIMAT0000063	XPO5	57510	Proteomics	18668040
hsa-let-7b-5p	MIMAT0000063	MIB1	57534	CLASH	23622248
hsa-let-7b-5p	MIMAT0000063	KIAA1328	57536	HITS-CLIP	23313552
hsa-let-7b-5p	MIMAT0000063	PDP2	57546	CLASH//PAR-CLIP	23622248|23592263
hsa-let-7b-5p	MIMAT0000063	CEP126	57562	CLASH	23622248
hsa-let-7b-5p	MIMAT0000063	ZNF687	57592	CLASH	23622248
hsa-let-7b-5p	MIMAT0000063	KIAA1549	57670	CLASH	23622248
hsa-let-7b-5p	MIMAT0000063	KIAA1586	57691	CLASH	23622248
hsa-let-7b-5p	MIMAT0000063	ZNF317	57693	CLASH	23622248

hsa-let-7b-5p	MIMAT0000063	IGDCC4	57722	PAR-CLIP	23446348|21572407|20371350|26701625
hsa-let-7b-5p	MIMAT0000063	NCOA5	57727	CLASH	23622248
hsa-let-7b-5p	MIMAT0000063	RAB40C	57799	PAR-CLIP	23592263
hsa-let-7b-5p	MIMAT0000063	RAP2C	57826	Proteomics	18668040
hsa-let-7b-5p	MIMAT0000063	TRAPPC1	58485	CLASH	23622248
hsa-let-7b-5p	MIMAT0000063	ABHD17C	58489	PAR-CLIP	20371350
hsa-let-7b-5p	MIMAT0000063	TLNRD1	59274	CLASH	23622248
hsa-let-7b-5p	MIMAT0000063	CACNG8	59283	CLASH	23622248
hsa-let-7b-5p	MIMAT0000063	SLC25A19	60386	Proteomics	18668040
hsa-let-7b-5p	MIMAT0000063	SPCS3	60559	Proteomics//pSILAC	18668040
hsa-let-7b-5p	MIMAT0000063	BCORL1	63035	CLASH	23622248
hsa-let-7b-5p	MIMAT0000063	GALNT11	63917	CLASH	23622248
hsa-let-7b-5p	MIMAT0000063	PRSS22	64063	HITS-CLIP	23313552
hsa-let-7b-5p	MIMAT0000063	XYLT2	64132	CLASH	23622248
hsa-let-7b-5p	MIMAT0000063	ERAP2	64167	CLASH	23622248
hsa-let-7b-5p	MIMAT0000063	DNAJC1	64215	Proteomics	18668040
hsa-let-7b-5p	MIMAT0000063	NSD1	64324	PAR-CLIP	24398324
hsa-let-7b-5p	MIMAT0000063	NXN	64359	Proteomics//pSILAC	18668040
hsa-let-7b-5p	MIMAT0000063	ZNF106	64397	CLASH	23622248
hsa-let-7b-5p	MIMAT0000063	GZF1	64412	Microarray	17699775
hsa-let-7b-5p	MIMAT0000063	NOM1	64434	PAR-CLIP	23592263|23446348
hsa-let-7b-5p	MIMAT0000063	CPEB1	64506	Luciferase reporter assay//Microarray//qRT-PCR	22995917
hsa-let-7b-5p	MIMAT0000063	ANAPC1	64682	Microarray//Proteomics//pSILAC//qRT-PCR//Western blot	18668040|26540468
hsa-let-7b-5p	MIMAT0000063	NUCKS1	64710	CLASH	23622248
hsa-let-7b-5p	MIMAT0000063	TBC1D15	64786	Proteomics	18668040
hsa-let-7b-5p	MIMAT0000063	ELOVL1	64834	Proteomics	18668040
hsa-let-7b-5p	MIMAT0000063	TUT1	64852	CLASH	23622248
hsa-let-7b-5p	MIMAT0000063	AIDA	64853	CLASH	23622248
hsa-let-7b-5p	MIMAT0000063	CCDC71	64925	CLASH	23622248
hsa-let-7b-5p	MIMAT0000063	NT5DC2	64943	CLASH	23622248
hsa-let-7b-5p	MIMAT0000063	MRPS24	64951	Proteomics//pSILAC	18668040
hsa-let-7b-5p	MIMAT0000063	MRPS11	64963	CLASH	23622248
hsa-let-7b-5p	MIMAT0000063	NOL6	65083	Proteomics	18668040

hsa-let-7b-5p	MIMAT0000063	MARCKSL1	65108	PAR-CLIP	26701625
hsa-let-7b-5p	MIMAT0000063	WNK1	65125	CLASH	23622248
hsa-let-7b-5p	MIMAT0000063	PYCR3	65263	Proteomics	18668040
hsa-let-7b-5p	MIMAT0000063	PLEKHA3	65977	PAR-CLIP	21572407
hsa-let-7b-5p	MIMAT0000063	PHACTR4	65979	HITS-CLIP	23706177
hsa-let-7b-5p	MIMAT0000063	GGCT	79017	Proteomics//pSILAC	18668040
hsa-let-7b-5p	MIMAT0000063	PDCL3	79031	CLASH	23622248
hsa-let-7b-5p	MIMAT0000063	NABP2	79035	CLASH	23622248
hsa-let-7b-5p	MIMAT0000063	KXD1	79036	CLASH	23622248
hsa-let-7b-5p	MIMAT0000063	ASPSCR1	79058	CLASH	23622248
hsa-let-7b-5p	MIMAT0000063	ATG9A	79065	PAR-CLIP//Proteomics	18668040|23592263|26701625|27292025
hsa-let-7b-5p	MIMAT0000063	FTO	79068	CLASH	23622248
hsa-let-7b-5p	MIMAT0000063	DCTPP1	79077	Proteomics//pSILAC	18668040
hsa-let-7b-5p	MIMAT0000063	ZNF426	79088	CLASH	23622248
hsa-let-7b-5p	MIMAT0000063	EFHD2	79180	PAR-CLIP	23592263
hsa-let-7b-5p	MIMAT0000063	WDR25	79446	CLASH	23622248
hsa-let-7b-5p	MIMAT0000063	ADIPOR2	79602	CLASH//PAR-CLIP	23622248|21572407
hsa-let-7b-5p	MIMAT0000063	RHBDF2	79651	PAR-CLIP	26701625
hsa-let-7b-5p	MIMAT0000063	MSANTD2	79684	CLASH	23622248
hsa-let-7b-5p	MIMAT0000063	IPO4	79711	CLASH//Proteomics//pSILAC	18668040|23622248
hsa-let-7b-5p	MIMAT0000063	LIN28A	79727	Luciferase reporter assay//Reporter assay	15131085
hsa-let-7b-5p	MIMAT0000063	NARS2	79731	Proteomics	18668040
hsa-let-7b-5p	MIMAT0000063	GEMIN7	79760	Proteomics//pSILAC	18668040
hsa-let-7b-5p	MIMAT0000063	ISOC2	79763	CLASH	23622248
hsa-let-7b-5p	MIMAT0000063	ZFHX4	79776	CLASH	23622248
hsa-let-7b-5p	MIMAT0000063	C12orf49	79794	CLASH	23622248
hsa-let-7b-5p	MIMAT0000063	NAA40	79829	Proteomics//pSILAC	18668040
hsa-let-7b-5p	MIMAT0000063	FAM57A	79850	CLASH	23622248
hsa-let-7b-5p	MIMAT0000063	CCDC134	79879	Proteomics	18668040
hsa-let-7b-5p	MIMAT0000063	MRM1	79922	Proteomics//pSILAC	18668040
hsa-let-7b-5p	MIMAT0000063	WLS	79971	Proteomics	18668040
hsa-let-7b-5p	MIMAT0000063	CPED1	79974	Microarray	17699775
hsa-let-7b-5p	MIMAT0000063	DOCK5	80005	Proteomics//pSILAC	18668040
hsa-let-7b-5p	MIMAT0000063	NAA25	80018	Proteomics//pSILAC	18668040
hsa-let-7b-5p	MIMAT0000063	FOXRED2	80020	CLASH	23622248

hsa-let-7b-5p	MIMAT0000063	ZNF556	80032	HITS-CLIP	23824327
hsa-let-7b-5p	MIMAT0000063	ZNF606	80095	CLASH	23622248
hsa-let-7b-5p	MIMAT0000063	PTGES2	80142	CLASH	23622248
hsa-let-7b-5p	MIMAT0000063	SIKE1	80143	CLASH	23622248
hsa-let-7b-5p	MIMAT0000063	NAA15	80155	Proteomics	18668040
hsa-let-7b-5p	MIMAT0000063	OPA3	80207	PAR-CLIP	23592263|26701625
hsa-let-7b-5p	MIMAT0000063	NAA50	80218	Proteomics	18668040
hsa-let-7b-5p	MIMAT0000063	PAAF1	80227	Proteomics	18668040
hsa-let-7b-5p	MIMAT0000063	WDR26	80232	CLASH	23622248
hsa-let-7b-5p	MIMAT0000063	EDEM3	80267	HITS-CLIP//Proteomics//pSILAC	18668040|23313552
hsa-let-7b-5p	MIMAT0000063	WDCP	80304	CLASH	23622248
hsa-let-7b-5p	MIMAT0000063	TRABD	80305	CLASH//Proteomics//pSILAC	18668040|23622248
hsa-let-7b-5p	MIMAT0000063	MED28	80306	CLASH	23622248
hsa-let-7b-5p	MIMAT0000063	FLAD1	80308	CLASH//Proteomics	18668040|23622248
hsa-let-7b-5p	MIMAT0000063	CPEB4	80315	Luciferase reporter assay//Microarray//qRT-PCR	22995917
hsa-let-7b-5p	MIMAT0000063	PUS1	80324	Proteomics	18668040
hsa-let-7b-5p	MIMAT0000063	REEP4	80346	Proteomics	18668040
hsa-let-7b-5p	MIMAT0000063	SLC19A3	80704	HITS-CLIP	23706177
hsa-let-7b-5p	MIMAT0000063	AARSD1	80755	Proteomics//pSILAC	18668040
hsa-let-7b-5p	MIMAT0000063	CPTP	80772	CLASH	23622248
hsa-let-7b-5p	MIMAT0000063	LIMD2	80774	PAR-CLIP	23592263
hsa-let-7b-5p	MIMAT0000063	INTS5	80789	Proteomics	18668040
hsa-let-7b-5p	MIMAT0000063	SLC25A32	81034	Proteomics//pSILAC	18668040
hsa-let-7b-5p	MIMAT0000063	COLEC12	81035	PAR-CLIP	21572407
hsa-let-7b-5p	MIMAT0000063	SLC38A1	81539	Proteomics	18668040
hsa-let-7b-5p	MIMAT0000063	GDPD5	81544	CLASH	23622248
hsa-let-7b-5p	MIMAT0000063	RCC1L	81554	Proteomics	18668040
hsa-let-7b-5p	MIMAT0000063	INTS14	81556	Proteomics	18668040
hsa-let-7b-5p	MIMAT0000063	C1orf21	81563	PAR-CLIP	20371350
hsa-let-7b-5p	MIMAT0000063	ANP32E	81611	Proteomics	18668040
hsa-let-7b-5p	MIMAT0000063	C6orf62	81688	CLASH	23622248
hsa-let-7b-5p	MIMAT0000063	ZNF611	81856	HITS-CLIP//PAR-CLIP	21572407|20371350|23706177
hsa-let-7b-5p	MIMAT0000063	MED25	81857	CLASH	23622248
hsa-let-7b-5p	MIMAT0000063	EMC6	83460	Proteomics	18668040

hsa-let-7b-5p	MIMAT0000063	DNAL1	83544	PAR-CLIP	21572407|27292025
hsa-let-7b-5p	MIMAT0000063	UCK1	83549	CLASH	23622248
hsa-let-7b-5p	MIMAT0000063	ATAD3B	83858	Proteomics//pSILAC	18668040
hsa-let-7b-5p	MIMAT0000063	CDCA7	83879	Microarray	17699775
hsa-let-7b-5p	MIMAT0000063	HASPIN	83903	HITS-CLIP//PAR-CLIP	21572407|20371350|23706177
hsa-let-7b-5p	MIMAT0000063	KREMEN1	83999	PAR-CLIP	23592263|26701625
hsa-let-7b-5p	MIMAT0000063	EMILIN2	84034	HITS-CLIP//PAR-CLIP	20371350|23706177
hsa-let-7b-5p	MIMAT0000063	SLC10A7	84068	PAR-CLIP	24398324|21572407|20371350
hsa-let-7b-5p	MIMAT0000063	WDR75	84128	Proteomics	18668040
hsa-let-7b-5p	MIMAT0000063	TOMM40L	84134	PAR-CLIP	26701625|27292025
hsa-let-7b-5p	MIMAT0000063	UTP15	84135	Proteomics	18668040
hsa-let-7b-5p	MIMAT0000063	ZNF644	84146	PAR-CLIP	23446348
hsa-let-7b-5p	MIMAT0000063	ANTXR1	84168	CLASH	23622248
hsa-let-7b-5p	MIMAT0000063	POLR1B	84172	Proteomics	18668040
hsa-let-7b-5p	MIMAT0000063	FAM96A	84191	Proteomics//pSILAC	18668040
hsa-let-7b-5p	MIMAT0000063	ZCCHC9	84240	CLASH	23622248
hsa-let-7b-5p	MIMAT0000063	NOA1	84273	PAR-CLIP	23592263|23446348
hsa-let-7b-5p	MIMAT0000063	FAM213A	84293	Proteomics	18668040
hsa-let-7b-5p	MIMAT0000063	LLPH	84298	Proteomics	18668040
hsa-let-7b-5p	MIMAT0000063	MIEN1	84299	CLASH	23622248
hsa-let-7b-5p	MIMAT0000063	CCDC115	84317	Proteomics//pSILAC	18668040
hsa-let-7b-5p	MIMAT0000063	LZIC	84328	Proteomics	18668040
hsa-let-7b-5p	MIMAT0000063	GFM2	84340	Proteomics	18668040
hsa-let-7b-5p	MIMAT0000063	CYSTM1	84418	CLASH	23622248
hsa-let-7b-5p	MIMAT0000063	RAB11FIP4	84440	PAR-CLIP	24398324
hsa-let-7b-5p	MIMAT0000063	ZBTB37	84614	PAR-CLIP	20371350
hsa-let-7b-5p	MIMAT0000063	USP38	84640	PAR-CLIP	21572407
hsa-let-7b-5p	MIMAT0000063	FAM126A	84668	CLASH	23622248
hsa-let-7b-5p	MIMAT0000063	GTPBP3	84705	Proteomics//pSILAC	18668040
hsa-let-7b-5p	MIMAT0000063	PRRC2B	84726	CLASH	23622248
hsa-let-7b-5p	MIMAT0000063	FUT10	84750	PAR-CLIP	23592263|26701625
hsa-let-7b-5p	MIMAT0000063	TUBA1C	84790	CLASH	23622248
hsa-let-7b-5p	MIMAT0000063	IL17RC	84818	CLASH	23622248
hsa-let-7b-5p	MIMAT0000063	PLXDC2	84898	Proteomics	18668040
hsa-let-7b-5p	MIMAT0000063	FAM136A	84908	CLASH	23622248
hsa-let-7b-5p	MIMAT0000063	ZNF587	84914	PAR-CLIP	23592263
hsa-let-7b-5p	MIMAT0000063	PPP1R15B	84919	PAR-CLIP	23592263|27292025

hsa-let-7b-5p	MIMAT0000063	FAM104A	84923	PAR-CLIP	20371350
hsa-let-7b-5p	MIMAT0000063	ZNF566	84924	PAR-CLIP	23446348|21572407|20371350
hsa-let-7b-5p	MIMAT0000063	MPND	84954	CLASH	23622248
hsa-let-7b-5p	MIMAT0000063	FBXL20	84961	PAR-CLIP	21572407
hsa-let-7b-5p	MIMAT0000063	REPS1	85021	CLASH	23622248
hsa-let-7b-5p	MIMAT0000063	HIST1H2BK	85236	PAR-CLIP	23592263|27292025
hsa-let-7b-5p	MIMAT0000063	ZCCHC3	85364	PAR-CLIP	23446348|26701625
hsa-let-7b-5p	MIMAT0000063	EAF1	85403	CLASH	23622248
hsa-let-7b-5p	MIMAT0000063	MIDN	90007	CLASH//PAR-CLIP	23622248|26701625
hsa-let-7b-5p	MIMAT0000063	ZNF799	90576	HITS-CLIP	23313552
hsa-let-7b-5p	MIMAT0000063	PIP4P1	90809	CLASH	23622248
hsa-let-7b-5p	MIMAT0000063	ADCK2	90956	CLASH	23622248
hsa-let-7b-5p	MIMAT0000063	DHX57	90957	CLASH//Proteomics//pSILAC	18668040|23622248
hsa-let-7b-5p	MIMAT0000063	FMNL3	91010	PAR-CLIP	22100165
hsa-let-7b-5p	MIMAT0000063	L3MBTL4	91133	CLASH	23622248
hsa-let-7b-5p	MIMAT0000063	YTHDC1	91746	CLASH	23622248
hsa-let-7b-5p	MIMAT0000063	NEK9	91754	CLASH	23622248
hsa-let-7b-5p	MIMAT0000063	C11orf52	91894	CLASH	23622248
hsa-let-7b-5p	MIMAT0000063	ZC3HAV1L	92092	PAR-CLIP	23592263
hsa-let-7b-5p	MIMAT0000063	OXNAD1	92106	Proteomics	18668040
hsa-let-7b-5p	MIMAT0000063	MTSS1L	92154	CLASH	23622248
hsa-let-7b-5p	MIMAT0000063	NAF1	92345	Proteomics	18668040
hsa-let-7b-5p	MIMAT0000063	MOB1B	92597	Proteomics	18668040
hsa-let-7b-5p	MIMAT0000063	TIMM50	92609	CLASH	23622248
hsa-let-7b-5p	MIMAT0000063	MGME1	92667	Proteomics//pSILAC	18668040
hsa-let-7b-5p	MIMAT0000063	SLC38A5	92745	Proteomics	18668040
hsa-let-7b-5p	MIMAT0000063	MARS2	92935	PAR-CLIP//pSILAC	18668040|26701625
hsa-let-7b-5p	MIMAT0000063	IGSF8	93185	CLASH	23622248
hsa-let-7b-5p	MIMAT0000063	SDR42E1	93517	PAR-CLIP	24398324
hsa-let-7b-5p	MIMAT0000063	ZFAND4	93550	HITS-CLIP//PAR-CLIP	23706177|23313552|27292025
hsa-let-7b-5p	MIMAT0000063	MFSD3	113655	CLASH	23622248
hsa-let-7b-5p	MIMAT0000063	TOE1	114034	Proteomics	18668040
hsa-let-7b-5p	MIMAT0000063	BTBD9	114781	CLASH	23622248
hsa-let-7b-5p	MIMAT0000063	MBD6	114785	CLASH	23622248
hsa-let-7b-5p	MIMAT0000063	OSBPL10	114884	Proteomics	18668040
hsa-let-7b-5p	MIMAT0000063	KCTD12	115207	Proteomics	18668040

hsa-let-7b-5p	MIMAT0000063	CTHRC1	115908	Luciferase reporter assay//qRT-PCR//Western blot	25510669
hsa-let-7b-5p	MIMAT0000063	TSEN15	116461	CLASH	23622248
hsa-let-7b-5p	MIMAT0000063	DCD	117159	Proteomics	18668040
hsa-let-7b-5p	MIMAT0000063	RFFL	117584	CLASH	23622248
hsa-let-7b-5p	MIMAT0000063	PDZD8	118987	PAR-CLIP	21572407
hsa-let-7b-5p	MIMAT0000063	LRIG3	121227	PAR-CLIP	21572407|20371350
hsa-let-7b-5p	MIMAT0000063	NAA30	122830	PAR-CLIP	21572407
hsa-let-7b-5p	MIMAT0000063	MSI2	124540	PAR-CLIP	21572407
hsa-let-7b-5p	MIMAT0000063	C19orf47	126526	PAR-CLIP	21572407|20371350
hsa-let-7b-5p	MIMAT0000063	GABPB2	126626	CLASH	23622248
hsa-let-7b-5p	MIMAT0000063	TBC1D20	128637	CLASH	23622248
hsa-let-7b-5p	MIMAT0000063	PIGU	128869	Proteomics	18668040
hsa-let-7b-5p	MIMAT0000063	NUP35	129401	Proteomics	18668040
hsa-let-7b-5p	MIMAT0000063	TRIM71	131405	PAR-CLIP	20371350
hsa-let-7b-5p	MIMAT0000063	FAM131A	131408	CLASH	23622248
hsa-let-7b-5p	MIMAT0000063	FAM43A	131583	PAR-CLIP	23592263|20371350
hsa-let-7b-5p	MIMAT0000063	GRPEL2	134266	CLASH//PAR-CLIP//Proteomics//pSILAC	18668040|23622248|23592263|24398324|23446348|21572407|20371350|26701625
hsa-let-7b-5p	MIMAT0000063	C5orf24	134553	CLASH	23622248
hsa-let-7b-5p	MIMAT0000063	PM20D2	135293	PAR-CLIP	21572407
hsa-let-7b-5p	MIMAT0000063	MTPN	136319	Reporter assay	15806104
hsa-let-7b-5p	MIMAT0000063	UBXN2B	137886	PAR-CLIP	21572407
hsa-let-7b-5p	MIMAT0000063	GPAT4	137964	PAR-CLIP	26701625
hsa-let-7b-5p	MIMAT0000063	BRI3BP	140707	HITS-CLIP	23706177
hsa-let-7b-5p	MIMAT0000063	SMCR8	140775	PAR-CLIP	24398324|23446348|21572407|26701625|27292025
hsa-let-7b-5p	MIMAT0000063	E2F7	144455	CLASH	23622248
hsa-let-7b-5p	MIMAT0000063	ZNF578	147660	HITS-CLIP	23706177
hsa-let-7b-5p	MIMAT0000063	ZNF417	147687	HITS-CLIP	23313552
hsa-let-7b-5p	MIMAT0000063	ZNF738	148203	HITS-CLIP	23706177
hsa-let-7b-5p	MIMAT0000063	SLC30A7	148867	Proteomics	18668040
hsa-let-7b-5p	MIMAT0000063	C1orf210	149466	HITS-CLIP//PAR-CLIP	23446348|21572407|23706177
hsa-let-7b-5p	MIMAT0000063	DTX3L	151636	HITS-CLIP	23706177
hsa-let-7b-5p	MIMAT0000063	CMC1	152100	Proteomics	18668040
hsa-let-7b-5p	MIMAT0000063	DAB2IP	153090	CLASH	23622248

hsa-let-7b-5p	MIMAT0000063	CEP120	153241	PAR-CLIP	23592263
hsa-let-7b-5p	MIMAT0000063	TMEM167A	153339	Proteomics	18668040
hsa-let-7b-5p	MIMAT0000063	TMEM65	157378	Proteomics	18668040
hsa-let-7b-5p	MIMAT0000063	RDH10	157506	Luciferase reporter assay//Proteomics//pSILAC	18668040
hsa-let-7b-5p	MIMAT0000063	ANKRD46	157567	PAR-CLIP	22100165|26701625
hsa-let-7b-5p	MIMAT0000063	FAM84B	157638	CLASH	23622248
hsa-let-7b-5p	MIMAT0000063	USP54	159195	CLASH	23622248
hsa-let-7b-5p	MIMAT0000063	TMTC3	160418	PAR-CLIP//Proteomics	18668040|23592263
hsa-let-7b-5p	MIMAT0000063	IFNLR1	163702	PAR-CLIP	23592263
hsa-let-7b-5p	MIMAT0000063	AGO3	192669	Microarray	17699775
hsa-let-7b-5p	MIMAT0000063	TMEM201	199953	CLASH//Proteomics	18668040|23622248
hsa-let-7b-5p	MIMAT0000063	TXLNA	200081	CLASH//PAR-CLIP	23622248|21572407
hsa-let-7b-5p	MIMAT0000063	IBA57	200205	CLASH	23622248
hsa-let-7b-5p	MIMAT0000063	KLHDC8B	200942	PAR-CLIP	23592263
hsa-let-7b-5p	MIMAT0000063	CENPV	201161	Proteomics	18668040
hsa-let-7b-5p	MIMAT0000063	UNC13D	201294	Proteomics	18668040
hsa-let-7b-5p	MIMAT0000063	ZNF584	201514	HITS-CLIP	23706177
hsa-let-7b-5p	MIMAT0000063	PDE12	201626	Microarray//PAR-CLIP//Proteomics	17699775|18668040|24398324|23446348|21572407|20371350
hsa-let-7b-5p	MIMAT0000063	TUBB	203068	CLASH	23622248
hsa-let-7b-5p	MIMAT0000063	HIPK1	204851	CLASH	23622248
hsa-let-7b-5p	MIMAT0000063	CCNY	219771	Proteomics	18668040
hsa-let-7b-5p	MIMAT0000063	SLC16A9	220963	PAR-CLIP	21572407
hsa-let-7b-5p	MIMAT0000063	FOXK1	221937	Proteomics	18668040
hsa-let-7b-5p	MIMAT0000063	TMED4	222068	PAR-CLIP	27292025
hsa-let-7b-5p	MIMAT0000063	SLC35F1	222553	CLASH	23622248
hsa-let-7b-5p	MIMAT0000063	AFG1L	246269	CLASH	23622248
hsa-let-7b-5p	MIMAT0000063	MMS22L	253714	Proteomics//pSILAC	18668040
hsa-let-7b-5p	MIMAT0000063	RNF144B	255488	PAR-CLIP	23592263
hsa-let-7b-5p	MIMAT0000063	ELMOD2	255520	CLASH	23622248
hsa-let-7b-5p	MIMAT0000063	MFSD8	256471	PAR-CLIP	24398324
hsa-let-7b-5p	MIMAT0000063	PGM2L1	283209	PAR-CLIP	23592263
hsa-let-7b-5p	MIMAT0000063	KCTD21	283219	PAR-CLIP	23592263
hsa-let-7b-5p	MIMAT0000063	TTC9C	283237	Proteomics//pSILAC	18668040
hsa-let-7b-5p	MIMAT0000063	ANKRD52	283373	CLASH	23622248
hsa-let-7b-5p	MIMAT0000063	SPRYD4	283377	Proteomics//pSILAC	18668040

hsa-let-7b-5p	MIMAT0000063	GATC	283459	Proteomics	18668040
hsa-let-7b-5p	MIMAT0000063	PRTG	283659	CLASH	23622248
hsa-let-7b-5p	MIMAT0000063	ZADH2	284273	CLASH//Proteomics	18668040|23622248
hsa-let-7b-5p	MIMAT0000063	ZNF841	284371	CLASH	23622248
hsa-let-7b-5p	MIMAT0000063	NBPF15	284565	CLASH	23622248
hsa-let-7b-5p	MIMAT0000063	C5orf51	285636	PAR-CLIP	23592263|24398324|23446348|21572407|20371350|26701625|27292025
hsa-let-7b-5p	MIMAT0000063	RNASE10	338879	CLASH	23622248
hsa-let-7b-5p	MIMAT0000063	ZBTB8OS	339487	HITS-CLIP//PAR-CLIP	23446348|23706177
hsa-let-7b-5p	MIMAT0000063	NAT8L	339983	PAR-CLIP	21572407
hsa-let-7b-5p	MIMAT0000063	ACER2	340485	PAR-CLIP	24398324
hsa-let-7b-5p	MIMAT0000063	ZNF774	342132	HITS-CLIP//PAR-CLIP	22100165|27418678
hsa-let-7b-5p	MIMAT0000063	ATXN1L	342371	CLASH	23622248
hsa-let-7b-5p	MIMAT0000063	MTX3	345778	HITS-CLIP	23706177
hsa-let-7b-5p	MIMAT0000063	SPATA12	353324	CLASH	23622248
hsa-let-7b-5p	MIMAT0000063	IRF2BP2	359948	CLASH	23622248
hsa-let-7b-5p	MIMAT0000063	NDUFA4P1	360165	PAR-CLIP	22100165
hsa-let-7b-5p	MIMAT0000063	NHLRC2	374354	HITS-CLIP	23313552
hsa-let-7b-5p	MIMAT0000063	DRAXIN	374946	CLASH	23622248
hsa-let-7b-5p	MIMAT0000063	NHLRC3	387921	PAR-CLIP	24398324|21572407|20371350
hsa-let-7b-5p	MIMAT0000063	BEND4	389206	PAR-CLIP	21572407|20371350
hsa-let-7b-5p	MIMAT0000063	PSMG4	389362	CLASH	23622248
hsa-let-7b-5p	MIMAT0000063	LIN28B	389421	Luciferase reporter assay//Microarray	17699775|16971064
hsa-let-7b-5p	MIMAT0000063	RBM12B	389677	PAR-CLIP//Proteomics	18668040|21572407|20371350
hsa-let-7b-5p	MIMAT0000063	NUDT19	390916	Proteomics//pSILAC	18668040
hsa-let-7b-5p	MIMAT0000063	ZNF805	390980	CLASH	23622248
hsa-let-7b-5p	MIMAT0000063	RAB19	401409	HITS-CLIP//PAR-CLIP	23706177|23313552|27292025
hsa-let-7b-5p	MIMAT0000063	POTEG	404785	CLASH//PAR-CLIP	23622248|26701625|27292025
hsa-let-7b-5p	MIMAT0000063	NOMO3	408050	CLASH	23622248
hsa-let-7b-5p	MIMAT0000063	FNDC9	408263	PAR-CLIP	26701625
hsa-let-7b-5p	MIMAT0000063	ATXN7L3B	552889	PAR-CLIP	23592263|20371350
hsa-let-7b-5p	MIMAT0000063	PRR5-ARHGAP8	553158	PAR-CLIP	27292025
hsa-let-7b-5p	MIMAT0000063	POTEM	641455	PAR-CLIP	26701625|27292025
hsa-let-7b-5p	MIMAT0000063	FAM83G	644815	PAR-CLIP	23592263|24398324
hsa-let-7b-5p	MIMAT0000063	ANXA8	653145	Proteomics	18668040

hsa-let-7b-5p	MIMAT0000063	ANXA8L1	728113	Proteomics	18668040
hsa-let-7b-5p	MIMAT0000063	CASTOR2	729438	PAR-CLIP	26701625
hsa-let-7b-5p	MIMAT0000063	POM121C	100101267	Proteomics	18668040
hsa-let-7b-5p	MIMAT0000063	TIMM23	100287932	Proteomics	18668040
hsa-let-7b-5p	MIMAT0000063	OCLN	100506658	CLASH	23622248
hsa-mir-22-3p	MIMAT0000077	ACLY	47	Luciferase reporter assay//qRT-PCR//Western blot	27317765
hsa-mir-22-3p	MIMAT0000077	AKT1	207	Luciferase reporter assay//Western blot	26364720
hsa-mir-22-3p	MIMAT0000077	BDNF	627	Luciferase reporter assay//Microarray	21168126
hsa-mir-22-3p	MIMAT0000077	BMP6	654	Luciferase reporter assay	24163368
hsa-mir-22-3p	MIMAT0000077	BMP7	655	Luciferase reporter assay//qRT-PCR//Western blot	19011694|24163368
hsa-mir-22-3p	MIMAT0000077	BMPR1B	658	Luciferase reporter assay	24163368
hsa-mir-22-3p	MIMAT0000077	BSG	682	Immunohistochemistry//Luciferase reporter assay//qRT-PCR//Western blot	24906624|28184176
hsa-mir-22-3p	MIMAT0000077	BTF3	689	Sequencing	20371350
hsa-mir-22-3p	MIMAT0000077	BTG1	694	Luciferase reporter assay	25449431
hsa-mir-22-3p	MIMAT0000077	BUB1B	701	CLASH	23622248
hsa-mir-22-3p	MIMAT0000077	CCNA2	890	Luciferase reporter assay	25596928
hsa-mir-22-3p	MIMAT0000077	CCNT2	905	PAR-CLIP//Sequencing	20371350|24398324
hsa-mir-22-3p	MIMAT0000077	CD151	977	Luciferase reporter assay//qRT-PCR//Western blot	24495805
hsa-mir-22-3p	MIMAT0000077	CDK6	1021	Sequencing	20371350
hsa-mir-22-3p	MIMAT0000077	CDKN1A	1026	Luciferase reporter assay//PAR-CLIP//qRT-PCR//Western blot	23582783|21572407
hsa-mir-22-3p	MIMAT0000077	CSF1R	1436	Luciferase reporter assay//qRT-PCR	24198819
hsa-mir-22-3p	MIMAT0000077	CSNK2A1	1457	Sequencing	20371350
hsa-mir-22-3p	MIMAT0000077	DAD1	1603	PAR-CLIP	27292025
hsa-mir-22-3p	MIMAT0000077	DDX6	1656	PAR-CLIP	22012620

hsa-mir-22-3p	MIMAT0000077	E2F2	1870	PAR-CLIP//Sequencing	20371350|26701625
hsa-mir-22-3p	MIMAT0000077	ERBB2	2064	Luciferase reporter assay	26544868
hsa-mir-22-3p	MIMAT0000077	ERBB3	2065	Luciferase reporter assay//qRT-PCR//Western blot	22484852
hsa-mir-22-3p	MIMAT0000077	ESR1	2099	Immunoblot//Luciferase reporter assay//qRT-PCR//Western blot	18347104|19414598
hsa-mir-22-3p	MIMAT0000077	MECOM	2122	5RACE//ChIP//Co-immunoprecipitation//Luciferase reporter assay//Northern blot//Western blot	27617961
hsa-mir-22-3p	MIMAT0000077	FKBP5	2289	CLASH	23622248
hsa-mir-22-3p	MIMAT0000077	GRB2	2885	PAR-CLIP	26701625
hsa-mir-22-3p	MIMAT0000077	NR3C1	2908	Sequencing	20371350
hsa-mir-22-3p	MIMAT0000077	H3F3B	3021	PAR-CLIP//Sequencing	20371350
hsa-mir-22-3p	MIMAT0000077	HIF1A	3091	Immunofluorescence//qRT-PCR//Western blot	24496460
hsa-mir-22-3p	MIMAT0000077	HMGB1	3146	Luciferase reporter assay//qRT-PCR//Western blot	23303785
hsa-mir-22-3p	MIMAT0000077	HSPA1B	3304	CLASH	23622248
hsa-mir-22-3p	MIMAT0000077	HTR2C	3358	Luciferase reporter assay//Microarray	21168126
hsa-mir-22-3p	MIMAT0000077	CYR61	3491	Luciferase reporter assay//qRT-PCR//Western blot	24449575
hsa-mir-22-3p	MIMAT0000077	CXCR2	3579	Flow//Gluc reporter assay//GUS reporter assay//HITS-CLIP//Immunoblot//Immunocytochemistry//Immunofluorescence//Immunohistochemistry//Immunoprecipitaion//Luciferase reporter assay//qRT-PCR//RNA immunoprecipitation assay (RIP)//Western blot	26364720

hsa-mir-22-3p	MIMAT0000077	INSIG1	3638	HITS-CLIP	19536157
hsa-mir-22-3p	MIMAT0000077	IRF5	3663	Luciferase reporter assay//qRT-PCR//Western blot	23303785
hsa-mir-22-3p	MIMAT0000077	RPSA	3921	CLASH	23622248
hsa-mir-22-3p	MIMAT0000077	LBP	3929	CLASH	23622248
hsa-mir-22-3p	MIMAT0000077	LGALS1	3956	Immunofluorescence//qRT-PCR//Western blot	24496460
hsa-mir-22-3p	MIMAT0000077	LGALS9	3965	Flow//Luciferase reporter assay//qRT-PCR//Western blot	26239725
hsa-mir-22-3p	MIMAT0000077	MAOA	4128	Luciferase reporter assay//Microarray	21168126
hsa-mir-22-3p	MIMAT0000077	MAX	4149	PAR-CLIP	23592263
hsa-mir-22-3p	MIMAT0000077	MMP14	4323	Luciferase reporter assay//Microarray//qRT-PCR//Western blot	26610210
hsa-mir-22-3p	MIMAT0000077	MTHFR	4524	Immunoblot//Luciferase reporter assay//qRT-PCR//Western blot	28045918
hsa-mir-22-3p	MIMAT0000077	MYO6	4646	CLASH	23622248
hsa-mir-22-3p	MIMAT0000077	NTRK2	4915	Luciferase reporter assay//qRT-PCR//Western blot	27662840
hsa-mir-22-3p	MIMAT0000077	PDHA1	5160	Sequencing	20371350
hsa-mir-22-3p	MIMAT0000077	PIK3C2A	5286	Sequencing	20371350
hsa-mir-22-3p	MIMAT0000077	PLK1	5347	Microarray//qRT-PCR//Western blot	25970317
hsa-mir-22-3p	MIMAT0000077	PPARA	5465	Luciferase reporter assay//qRT-PCR//Western blot	18347104|19011694
hsa-mir-22-3p	MIMAT0000077	PRKACA	5566	Sequencing	20371350
hsa-mir-22-3p	MIMAT0000077	PTEN	5728	Luciferase reporter assay	20388916|26544868
hsa-mir-22-3p	MIMAT0000077	PTMS	5763	Luciferase reporter assay//Microarray//Northern blot//qRT-PCR//Western blot	22493679
hsa-mir-22-3p	MIMAT0000077	PEX5	5830	CLASH	23622248

hsa-mir-22-3p	MIMAT0000077	RAB5B	5869	Chromatin immunoprecipitation//FACS//Immunohistochemistry//Luciferase reporter assay//Microarray//Next Generation Sequencing (NGS)//Northern blot//PAR-CLIP//qRT-PCR//Sequencing//Western blot	20371350|21572407|27569217
hsa-mir-22-3p	MIMAT0000077	RAP2B	5912	HITS-CLIP	23313552
hsa-mir-22-3p	MIMAT0000077	RBL1	5933	PAR-CLIP	20371350
hsa-mir-22-3p	MIMAT0000077	RGS2	5997	Luciferase reporter assay//Microarray	21168126|23349832
hsa-mir-22-3p	MIMAT0000077	RPL24	6152	CLASH	23622248
hsa-mir-22-3p	MIMAT0000077	RPL35A	6165	CLASH	23622248
hsa-mir-22-3p	MIMAT0000077	RPS2	6187	CLASH	23622248
hsa-mir-22-3p	MIMAT0000077	RPS4X	6191	CLASH	23622248
hsa-mir-22-3p	MIMAT0000077	SCD	6319	HITS-CLIP	23313552
hsa-mir-22-3p	MIMAT0000077	SRSF7	6432	Sequencing	20371350
hsa-mir-22-3p	MIMAT0000077	SLC2A1	6513	HITS-CLIP//In situ hybridization//Luciferase reporter assay//qRT-PCR//Western blot	23313552|25304371
hsa-mir-22-3p	MIMAT0000077	SNAI1	6615	Luciferase reporter assay//Microarray//qRT-PCR//Western blot	26610210
hsa-mir-22-3p	MIMAT0000077	SP1	6667	Luciferase reporter assay//qRT-PCR//Reporter assay//Western blot	23529765|21502362|27904693
hsa-mir-22-3p	MIMAT0000077	SRPK1	6732	PAR-CLIP	20371350
hsa-mir-22-3p	MIMAT0000077	STX4	6810	CLASH	23622248

hsa-mir-22-3p	MIMAT0000077	TACC1	6867	Chromatin immunoprecipitation//FACS//Immunohistochemistry//Luciferase reporter assay//Microarray//Next Generation Sequencing (NGS)//Northern blot//qRT-PCR//Western blot	27569217
hsa-mir-22-3p	MIMAT0000077	TBX3	6926	Sequencing	20371350
hsa-mir-22-3p	MIMAT0000077	TCF7	6932	//Luciferase reporter assay//qRT-PCR//Western blot	26193896
hsa-mir-22-3p	MIMAT0000077	TFRC	7037	flow//Luciferase reporter assay//Northern blot	19135902
hsa-mir-22-3p	MIMAT0000077	TIAM1	7074	Luciferase reporter assay//Western blot	23440286
hsa-mir-22-3p	MIMAT0000077	TPD52L2	7165	PAR-CLIP	23592263
hsa-mir-22-3p	MIMAT0000077	VSNL1	7447	HITS-CLIP//PAR-CLIP	21572407|20371350|23824327|27418678
hsa-mir-22-3p	MIMAT0000077	WNT1	7471	Immunohistochemistry//Luciferase reporter assay//Microarray//qRT-PCR//Western blot	23851184
hsa-mir-22-3p	MIMAT0000077	YWHAZ	7534	PAR-CLIP	24398324|23446348|20371350
hsa-mir-22-3p	MIMAT0000077	ZNF217	7764	Sequencing	20371350
hsa-mir-22-3p	MIMAT0000077	ALMS1	7840	CLASH	23622248
hsa-mir-22-3p	MIMAT0000077	NUP214	8021	CLASH	23622248
hsa-mir-22-3p	MIMAT0000077	SLC7A5	8140	PAR-CLIP	26701625
hsa-mir-22-3p	MIMAT0000077	NCOA1	8648	Luciferase reporter assay//qRT-PCR//Western blot	21798241|26244872
hsa-mir-22-3p	MIMAT0000077	TNFRSF10D	8793	PAR-CLIP	22012620
hsa-mir-22-3p	MIMAT0000077	FUBP1	8880	CLASH	23622248
hsa-mir-22-3p	MIMAT0000077	MTA1	9112	Immunofluorescence//Immunohistochemistry//Luciferase reporter assay//qRT-PCR	28231399
hsa-mir-22-3p	MIMAT0000077	VAPB	9217	CLASH	23622248

hsa-mir-22-3p	MIMAT0000077	TCEAL1	9338	Immunoblot//Luciferase reporter assay//qRT-PCR	21565979
hsa-mir-22-3p	MIMAT0000077	RBM39	9584	CLASH	23622248
hsa-mir-22-3p	MIMAT0000077	ZNF646	9726	PAR-CLIP	22100165|22291592
hsa-mir-22-3p	MIMAT0000077	IFT140	9742	PAR-CLIP	20371350
hsa-mir-22-3p	MIMAT0000077	HDAC4	9759	Luciferase reporter assay//qRT-PCR//Western blot	20842113|23349832
hsa-mir-22-3p	MIMAT0000077	HDAC6	10013	Luciferase reporter assay//qRT-PCR//Western blot	22375943|28195408
hsa-mir-22-3p	MIMAT0000077	ARPC5	10092	EMSA//Immunohistochemistry//Luciferase reporter assay//Microarray//qRT-PCR//Western blot	22447776
hsa-mir-22-3p	MIMAT0000077	NET1	10276	Luciferase reporter assay//qRT-PCR//Western blot	25041463
hsa-mir-22-3p	MIMAT0000077	BTN3A3	10384	PAR-CLIP	20371350
hsa-mir-22-3p	MIMAT0000077	ZNF460	10794	PAR-CLIP	23592263
hsa-mir-22-3p	MIMAT0000077	EFR3B	22979	HITS-CLIP	19536157
hsa-mir-22-3p	MIMAT0000077	RCOR1	23186	Luciferase reporter assay	23349832
hsa-mir-22-3p	MIMAT0000077	TBC1D12	23232	Sequencing	20371350
hsa-mir-22-3p	MIMAT0000077	WWC1	23286	PAR-CLIP	22012620
hsa-mir-22-3p	MIMAT0000077	FRAT2	23401	PAR-CLIP//Sequencing	20371350|21572407
hsa-mir-22-3p	MIMAT0000077	SIRT1	23411	Luciferase reporter assay//qRT-PCR//Western blot	26912776|26662303
hsa-mir-22-3p	MIMAT0000077	TTC33	23548	HITS-CLIP	19536157
hsa-mir-22-3p	MIMAT0000077	ELP5	23587	CLASH	23622248
hsa-mir-22-3p	MIMAT0000077	LEMD3	23592	Sequencing	20371350
hsa-mir-22-3p	MIMAT0000077	ARHGEF26	26084	PAR-CLIP	27292025
hsa-mir-22-3p	MIMAT0000077	SERBP1	26135	PAR-CLIP	23446348
hsa-mir-22-3p	MIMAT0000077	TRAF3IP1	26146	HITS-CLIP	19536157
hsa-mir-22-3p	MIMAT0000077	MYCBP	26292	Immunoblot//Luciferase reporter assay//qRT-PCR	20562918
hsa-mir-22-3p	MIMAT0000077	FOXP1	27086	Sequencing	20371350
hsa-mir-22-3p	MIMAT0000077	PIGP	51227	HITS-CLIP	23824327

hsa-mir-22-3p	MIMAT0000077	UBR5	51366	qRT-PCR//Western blot	27124677
hsa-mir-22-3p	MIMAT0000077	GINS2	51659	HITS-CLIP	23824327
hsa-mir-22-3p	MIMAT0000077	CYCS	54205	HITS-CLIP	23824327
hsa-mir-22-3p	MIMAT0000077	DDIT4	54541	PAR-CLIP	23592263|21572407
hsa-mir-22-3p	MIMAT0000077	TET2	54790	Flow//Immunofluorescence//Immunohistochemistry//In situ hybridization//Luciferase reporter assay//qRT-PCR//Western blot	23830207|23827711
hsa-mir-22-3p	MIMAT0000077	DCAF16	54876	HITS-CLIP	23313552
hsa-mir-22-3p	MIMAT0000077	LRRC20	55222	HITS-CLIP	19536157
hsa-mir-22-3p	MIMAT0000077	LRRC1	55227	Sequencing	20371350
hsa-mir-22-3p	MIMAT0000077	MIS18BP1	55320	Sequencing	20371350
hsa-mir-22-3p	MIMAT0000077	LIN7C	55327	Sequencing	20371350
hsa-mir-22-3p	MIMAT0000077	CAMK2N1	55450	HITS-CLIP	23313552
hsa-mir-22-3p	MIMAT0000077	RCC2	55920	HITS-CLIP	23313552
hsa-mir-22-3p	MIMAT0000077	ZMAT5	55954	PAR-CLIP	23592263
hsa-mir-22-3p	MIMAT0000077	RBSN	64145	Sequencing	20371350
hsa-mir-22-3p	MIMAT0000077	RMND5A	64795	PAR-CLIP	24398324
hsa-mir-22-3p	MIMAT0000077	PHACTR4	65979	PAR-CLIP	26701625
hsa-mir-22-3p	MIMAT0000077	EDC3	80153	Sequencing	20371350
hsa-mir-22-3p	MIMAT0000077	CTC1	80169	PAR-CLIP	23446348|20371350
hsa-mir-22-3p	MIMAT0000077	CHD9	80205	HITS-CLIP	23313552
hsa-mir-22-3p	MIMAT0000077	SPG11	80208	CLASH	23622248
hsa-mir-22-3p	MIMAT0000077	CLPTM1L	81037	CLASH	23622248
hsa-mir-22-3p	MIMAT0000077	TSC22D4	81628	Sequencing	20371350
hsa-mir-22-3p	MIMAT0000077	LONP2	83752	HITS-CLIP	23824327|27418678
hsa-mir-22-3p	MIMAT0000077	KCTD10	83892	PAR-CLIP	23592263|24398324|26701625
hsa-mir-22-3p	MIMAT0000077	ARID5B	84159	Sequencing	20371350
hsa-mir-22-3p	MIMAT0000077	GLIS2	84662	CLASH	23622248
hsa-mir-22-3p	MIMAT0000077	MTDH	92140	Luciferase reporter assay//qRT-PCR//Western blot	25323629
hsa-mir-22-3p	MIMAT0000077	SFXN1	94081	CLASH	23622248
hsa-mir-22-3p	MIMAT0000077	VASN	114990	HITS-CLIP	19536157
hsa-mir-22-3p	MIMAT0000077	KCTD12	115207	CLASH	23622248
hsa-mir-22-3p	MIMAT0000077	C15orf40	123207	PAR-CLIP	23446348|27292025

hsa-mir-22-3p	MIMAT0000077	C1orf87	127795	HITS-CLIP	23824327
hsa-mir-22-3p	MIMAT0000077	ACVR1C	130399	Luciferase reporter assay//Reporter assay//Western blot	21224400
hsa-mir-22-3p	MIMAT0000077	C5orf24	134553	PAR-CLIP//Sequencing	20371350|23592263|24398324|23446348|21572407
hsa-mir-22-3p	MIMAT0000077	SOGA1	140710	Sequencing	20371350
hsa-mir-22-3p	MIMAT0000077	DNHD1	144132	CLASH	23622248
hsa-mir-22-3p	MIMAT0000077	TMEM120B	144404	HITS-CLIP	23706177
hsa-mir-22-3p	MIMAT0000077	PDIK1L	149420	PAR-CLIP	21572407
hsa-mir-22-3p	MIMAT0000077	PPM1K	152926	Luciferase reporter assay//qRT-PCR//Western blot	26592513
hsa-mir-22-3p	MIMAT0000077	PRELID2	153768	PAR-CLIP	22291592
hsa-mir-22-3p	MIMAT0000077	ZNF431	170959	PAR-CLIP	27292025
hsa-mir-22-3p	MIMAT0000077	TMEM201	199953	PAR-CLIP	26701625
hsa-mir-22-3p	MIMAT0000077	IBA57	200205	PAR-CLIP	26701625
hsa-mir-22-3p	MIMAT0000077	HNRNPA3	220988	HITS-CLIP	19536157
hsa-mir-22-3p	MIMAT0000077	FOXK1	221937	PAR-CLIP	26701625
hsa-mir-22-3p	MIMAT0000077	TMED4	222068	PAR-CLIP	24398324|21572407|20371350
hsa-mir-22-3p	MIMAT0000077	BRWD3	254065	PAR-CLIP	23592263
hsa-mir-22-3p	MIMAT0000077	MALAT1	378938	Flow//Gluc reporter assay//GUS reporter assay//HITS-CLIP//Immunoblot//Immunocytochemistry//Immunofluorescence//Immunohistochemistry//Immunoprecipitaion//Luciferase reporter assay//qRT-PCR//RNA immunoprecipitation assay (RIP)//Western blot	26364720
hsa-mir-22-3p	MIMAT0000077	ZNF662	389114	PAR-CLIP	20371350|27292025
hsa-mir-22-3p	MIMAT0000077	RAB44	401258	PAR-CLIP	22012620
hsa-mir-22-3p	MIMAT0000077	TMEM178B	100507421	HITS-CLIP	23824327|27418678
hsa-mir-184	MIMAT0000454	AKT1	207	Luciferase reporter assay	27666871

hsa-mir-184	MIMAT0000454	AKT2	208	Luciferase reporter assay//PAR-CLIP//qRT-PCR//Western blot	20409325|20371350|27418134
hsa-mir-184	MIMAT0000454	ARHGDIA	396	PAR-CLIP	26701625
hsa-mir-184	MIMAT0000454	BCL2	596	Luciferase reporter assay	24157866
hsa-mir-184	MIMAT0000454	BCL2L1	598	PAR-CLIP	26701625
hsa-mir-184	MIMAT0000454	TPP1	1200	PAR-CLIP	27292025
hsa-mir-184	MIMAT0000454	CSF1	1435	PAR-CLIP	20371350
hsa-mir-184	MIMAT0000454	GAS1	2619	qRT-PCR//Western blot	26805687
hsa-mir-184	MIMAT0000454	INPPL1	3636	Luciferase reporter assay//Northern blot//Western blot	19033458|24183204
hsa-mir-184	MIMAT0000454	LIFR	3977	HITS-CLIP	23313552
hsa-mir-184	MIMAT0000454	MEIS3P1	4213	PAR-CLIP	20371350
hsa-mir-184	MIMAT0000454	MYC	4609	Luciferase reporter assay	24157866
hsa-mir-184	MIMAT0000454	NFATC2	4773	Luciferase reporter assay//qRT-PCR//Western blot	19286996
hsa-mir-184	MIMAT0000454	NFIC	4782	PAR-CLIP	23592263|20371350|26701625|27292025
hsa-mir-184	MIMAT0000454	OPRD1	4985	PAR-CLIP	20371350
hsa-mir-184	MIMAT0000454	PDGFB	5155	Immunoblot//Luciferase reporter assay//qRT-PCR	27825105
hsa-mir-184	MIMAT0000454	PKM	5315	Immunocytochemistry//Luciferase reporter assay//qRT-PCR//Western blot	27431728
hsa-mir-184	MIMAT0000454	PLAGL2	5326	PAR-CLIP	26701625
hsa-mir-184	MIMAT0000454	PTPA	5524	PAR-CLIP	26701625
hsa-mir-184	MIMAT0000454	PRKCB	5579	Luciferase reporter assay	27666871
hsa-mir-184	MIMAT0000454	FSCN1	6624	PAR-CLIP	26701625
hsa-mir-184	MIMAT0000454	SURF6	6838	PAR-CLIP	26701625
hsa-mir-184	MIMAT0000454	TNFAIP2	7127	Immunohistochemistry//Luciferase reporter assay//qRT-PCR//Western blot	25888093
hsa-mir-184	MIMAT0000454	EZR	7430	Luciferase reporter assay//qRT-PCR//Western blot	25251993

hsa-mir-184	MIMAT0000454	CNBP	7555	PAR-CLIP	23592263|20371350
hsa-mir-184	MIMAT0000454	SLC7A5	8140	PAR-CLIP	23592263|24398324|22012620|26701625
hsa-mir-184	MIMAT0000454	PLPP3	8613	Immunoblot//Luciferase reporter assay//qRT-PCR	27825105
hsa-mir-184	MIMAT0000454	TM9SF4	9777	PAR-CLIP	26701625
hsa-mir-184	MIMAT0000454	CARM1	10498	PAR-CLIP	26701625|27292025
hsa-mir-184	MIMAT0000454	RAI1	10743	PAR-CLIP	23592263|20371350
hsa-mir-184	MIMAT0000454	PPP1R13L	10848	Luciferase reporter assay	28012196
hsa-mir-184	MIMAT0000454	PPP6R1	22870	PAR-CLIP	26701625
hsa-mir-184	MIMAT0000454	ZFPM2	23414	Immunoblot//Luciferase reporter assay//qRT-PCR	27825105
hsa-mir-184	MIMAT0000454	SND1	27044	Immunofluorescence//Immunohistochemistry//Luciferase reporter assay//qRT-PCR//Western blot	25216670
hsa-mir-184	MIMAT0000454	TJP3	27134	PAR-CLIP	23592263|20371350
hsa-mir-184	MIMAT0000454	AGO2	27161	Luciferase reporter assay//PAR-CLIP//Western blot	24361012|23696368|26701625
hsa-mir-184	MIMAT0000454	DESI1	27351	PAR-CLIP	22291592
hsa-mir-184	MIMAT0000454	TNPO2	30000	PAR-CLIP	23592263|24398324|22012620|21572407|20371350|26701625|27292025
hsa-mir-184	MIMAT0000454	TACO1	51204	HITS-CLIP	23313552
hsa-mir-184	MIMAT0000454	GNL3L	54552	HITS-CLIP	19536157
hsa-mir-184	MIMAT0000454	SELENOS	55829	HITS-CLIP//PAR-CLIP	22012620|20371350|23313552
hsa-mir-184	MIMAT0000454	BIN3	55909	Luciferase reporter assay	27666871
hsa-mir-184	MIMAT0000454	LRRC8A	56262	PAR-CLIP	20371350
hsa-mir-184	MIMAT0000454	CBX8	57332	PAR-CLIP	26701625
hsa-mir-184	MIMAT0000454	FN3K	64122	PAR-CLIP	23592263
hsa-mir-184	MIMAT0000454	KLC2	64837	PAR-CLIP	26701625
hsa-mir-184	MIMAT0000454	SOX7	83595	Flow//Luciferase reporter assay//qRT-PCR//Western blot	24558429
hsa-mir-184	MIMAT0000454	GPRIN1	114787	PAR-CLIP	23592263
hsa-mir-184	MIMAT0000454	TNFRSF13C	115650	HITS-CLIP	23313552
hsa-mir-184	MIMAT0000454	USH1G	124590	PAR-CLIP	24398324|20371350

hsa-mir-184	MIMAT0000454	IFFO2	126917	PAR-CLIP	26701625
hsa-mir-184	MIMAT0000454	ZSCAN25	221785	PAR-CLIP	26701625
hsa-mir-184	MIMAT0000454	CEP170B	283638	PAR-CLIP	26701625
hsa-mir-184	MIMAT0000454	PEAK3	374872	PAR-CLIP	27292025
hsa-mir-184	MIMAT0000454	POM121 C	100101 267	PAR-CLIP	23592263

Supplemental table 3. List of miRNA protein targets from MIRNET analyses
Experimental methods used to identify protein targets and the PMID of the related reports are indicated in the last two columns.
